# Unveiling the novel regulatory roles of RpoD-family sigma factors in *Salmonella* Typhimurium heat shock response through systems biology approaches

**DOI:** 10.1371/journal.pgen.1011464

**Published:** 2024-10-29

**Authors:** Joon Young Park, Minchang Jang, Sang-Mok Lee, Jihoon Woo, Eun-Jin Lee, Donghyuk Kim

**Affiliations:** 1 School of Energy and Chemical Engineering, Ulsan National Institute of Science and Technology (UNIST), Ulsan, Republic of Korea; 2 Department of Life Sciences, College of Life Sciences and Biotechnology, Korea University, Seoul, Republic of Korea; The University of Texas Southwestern Medical Center, UNITED STATES OF AMERICA

## Abstract

Three RpoD-family sigma factors, RpoD, RpoS, and RpoH, play critical roles in transcriptional regulation in *Salmonella enterica* serovar Typhimurium under heat shock conditions. However, the genome-wide regulatory mechanisms of these sigma factors in response to heat stress have remained elusive. In this study, we comprehensively identified 2,319, 2,226, and 213 genome-wide binding sites for RpoD, RpoS, and RpoH, respectively, under sublethal heat shock conditions (42°C). Machine learning-based transcriptome analysis was employed to infer the relative activity of iModulons, providing valuable insights into the transcriptional impact of heat shock. Integrative data analysis enabled the reconstruction of the transcriptional regulatory network of sigma factors, revealing how they modulate gene expression to adapt to heat stress, including responses to anaerobic and oxidative stresses. Notably, we observed a significant expansion of the RpoS sigmulon from 97 to 301 genes in response to heat shock, underscoring the crucial role of RpoS in regulating various metabolic processes. Moreover, we uncovered a competition mechanism between RpoD and RpoS within RpoS sigmulons, where RpoS significantly increases its binding within promoter regions shared with RpoD under heat shock conditions. These findings illuminate how three RpoD-family sigma factors coordinate multiple cellular processes to orchestrate the overall response of *S*. Typhimurium to heat stress.

## Author summary

RpoD-family sigma factors in *Salmonella enterica* serovar Typhimurium orchestrate transcription initiation for diverse metabolic genes in response to environmental stimuli. Heat stress, a fundamental challenge for bacteria in both natural and industrial settings. Typically triggers the up-regulation of heat shock proteins that assist in protein folding and degradation of damaged proteins. However, the full scope of transcriptional changes induced by heat shock extends beyond this familiar response. In this study, we employed systems biology approaches to elucidate detailed alterations in transcriptional regulatory networks (TRNs) mediated by RpoD-family sigma factors in response to heat shock. Machine learning-based transcriptomic analysis broke down the components of heat stress, revealing its involvement in both anaerobic and oxidative stress responses. By integrating genome-scale data with this nuanced understanding of heat shock, we reconstructed the TRNs and uncovered several key findings: (i) A competition mechanism exists between RpoS and RpoD at overlapping binding sites upstream of promoter region in response to heat shock; (ii) Heat-induced anaerobic stress down-regulated the majority of electron transporter genes, leading to significant modifications in the TRN governing carbon metabolism; (iii) Under heat shock conditions, alterations in iron availability induced down-regulation of extracellular iron uptake and enterobactin systems, while up-regulating intracellular iron-utilizing genes. This regulatory response may reduce intracellular iron concentrations, potentially attenuating Fenton reaction-mediated oxidative stress. These comprehensive results illuminate how RpoD-family sigma factors in *S*. Typhimurium regulate diverse cellular processes to adapt to heat shock, encompassing responses to anaerobic and oxidative stress. Our findings provide a more holistic understanding of bacterial heat shock response, extending beyond the classical heat shock protein paradigm.

## Introduction

*Salmonella enterica* serovar Typhimurium 14028s is a nontyphoidal Gram-negative pathogen that is a major cause of worldwide food-borne gastroenteritis, exhibiting proliferation across a diverse range of temperature conditions [1,2]. This remarkable thermal adaptability suggests that this pathogen possesses flexible transcriptional regulatory network (TRN), enabling the organism to adapt to fluctuating temperature conditions, particularly in response to heat stress. Heat treatment is widely utilized as an efficacious method for pathogen inactivation; however, sublethal heat exposure can potentially induce alterations in the TRNs of *S*. Typhimurium, potentially conferring enhanced resistance to diverse stress conditions [3,4]. Therefore, a detailed understanding of these changes in TRNs in response to heat stress is crucial for developing new therapeutic and preventive strategies to nontyphoidal salmonellosis.

Three representative RpoD-family sigma factors, RpoD, RpoS, and RpoH, play crucial roles in transcriptional regulation by binding to promoter regions and recruiting RNA polymerase (RNAP) to initiate transcription of most genes. These sigma factors adjust their binding affinities to regulate gene expression in response to environmental stimuli [5]. Therefore, identifying their binding profiles and changes in binding intensity is essential for understanding the TRNs of bacteria under heat stress. Various studies have examined the transcriptional regulation of *Salmonella*, highlighting the importance of sigma factors [4,6–9].

RpoD (σ^70^) is the primary housekeeping sigma factor responsible for expressing the majority of genes required for exponential growth under normal cellular conditions. Environmental changes alter the levels of alternative sigma factors within the cell, leading to their interaction with the core RNAP [9]. This interaction results in the redirection of RNAP, allowing for the selective expression of discrete gene subsets. Among the RpoD-family sigma factors, RpoS (σ^38^) serves as a global regulator, orchestrating gene expression in response to multiple stresses, from the stationary phase to various external conditions encountered by *S*. Typhimurium, such as inside of the infected host [10–13]. In particular, RpoS is vital in controlling stresses during the stationary phase of *Salmonella* and *E*. *coli*. These stresses include nutrient depletion [14–16], heat shock [17,18], oxidative stress [14,19], and acidic condition [20]. RpoS also regulates gene expression necessary for virulence in *S*. Typhimurium, which is essential for its proliferation within hosts [14,21–23]. While RpoS predominantly acts as a positive regulator, it also exhibits negative regulatory effect on the expression of some genes [5,24,25]. This negative effect is mainly found in overlapping promoter regions between RpoD and RpoS [5]. Competition between both these sigma factors leads to the repression of gene expression due to the lower recruitment activity of the RNAP core enzyme by RpoS compared to RpoD [5]. RpoH (σ^32^) is a key regulator of the heat shock response, controlling the expression of essential heat shock-related genes when temperatures rise [26]. The interaction of RpoH with RNAP core enzyme is tightly controlled by DnaK and DnaJ under non-heat stress conditions [27,28].

Previous studies have characterized the binding sites of sigma factors in *S*. Typhimurium 14028s using *in vitro* DNA-binding experiments and ChIP-methods with lower resolution, such as ChIP-chip and ChIP-seq [8,29,30]. However, the fluctuations in the binding profiles of RpoD-family sigma factors and their TRNs in response to heat shock have been studied only to a limited extent. In this study, genome-wide binding sites of three sigma factors were identified with near-1bp resolution under both control (37°C) and sublethal heat shock conditions (42°C) using ChIP-mini, a minimization method of ChIP-exo (Chromatin Immunoprecipitation with exonuclease treatment) that employs λ exonucleases [31]. In addition, transcription profiling (RNA-seq) was performed with *S*. Typhimurium wild-type and *ΔrpoS* strains to combine sigma factors binding profiles, revealing transcriptional regulatory mechanisms in response to heat shock. Furthermore, transcription profiling was applied to iModulons, which represent independently regulated groups of genes deconvoluted by independent component analysis (ICA), to decompose transcriptional impact of heat stress by inferring their relative activity [32,33].

## Results

### Reconstruction of *Salmonella* transcriptional regulatory networks in response to heat shock

**Fig 1:**
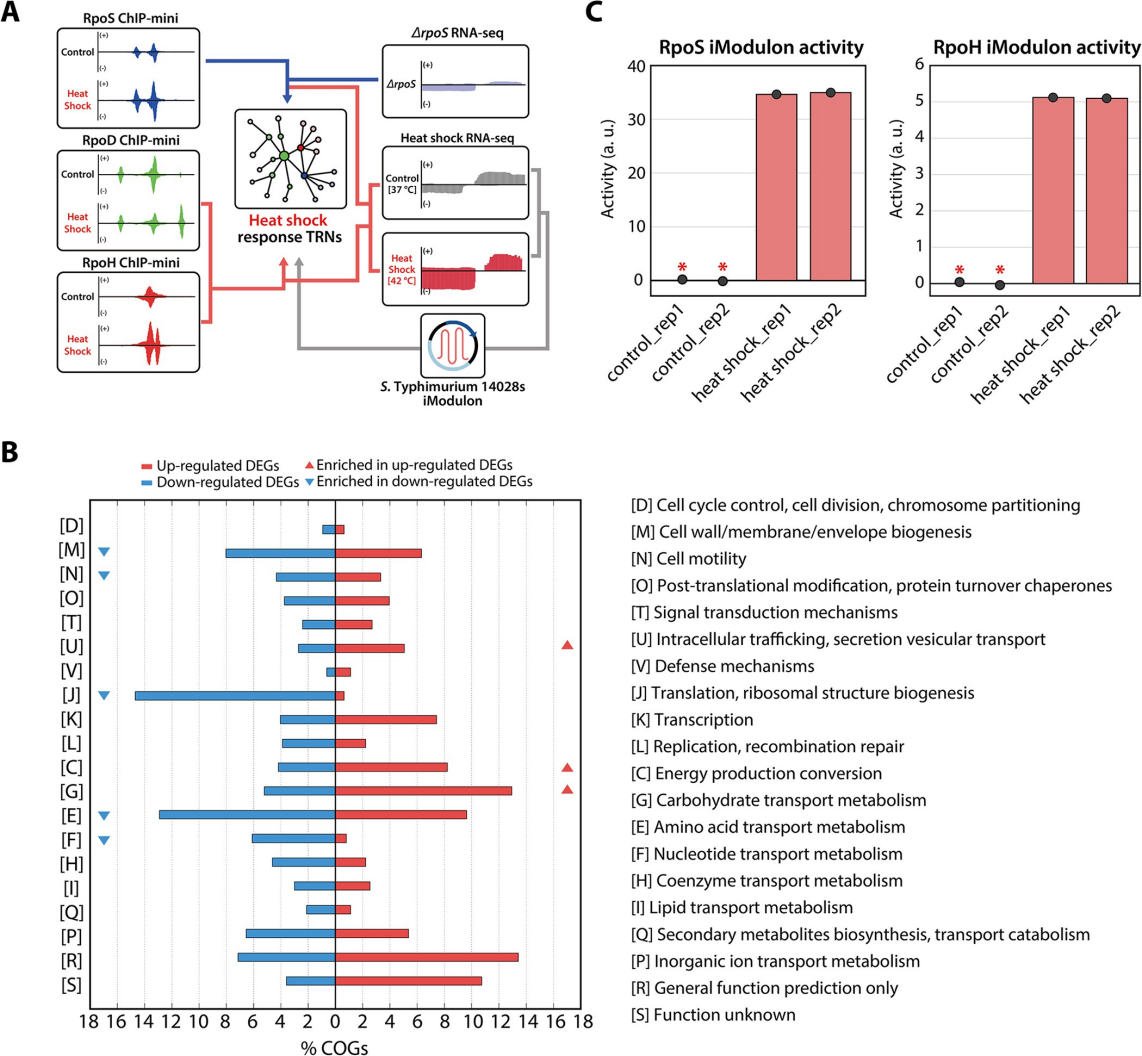
Overview of the reconstruction of *Salmonella* transcriptional regulatory network (TRN). **(**A) Schematic representation of TRN reconstruction for the heat shock response in *S*. Typhimurium 14028s. RpoS binding sites and transcript expression profiles of the *ΔrpoS* mutant are used to define the RpoS sigmulons, representing the set of genes directly regulated by RpoS. Binding sites of RpoD and RpoH, along with their expression changes upon heat shock, are used to establish both sigmulons. Additionally, a strain-specific RNA-seq compendium for *S*. Typhimurium 14028s is utilized to infer the activity levels of each iModulon, guiding predictions of changes in TRNs in response to heat shock. (B) COG analysis on up- and down-regulated differentially expressed genes (DEGs) in response to heat shock. Up-regulated DEGs have functions enriched in “Intracellular trafficking, secretion vesicular transport”, “Energy production conversion”, and “Carbohydrate transport metabolism” categories (Hypergeometric test *p*-value < 0.05). Down-regulated DEGs have functions enriched in “Cell wall/membrane/envelope biogenesis, “Cell motility”, “Translation, ribosomal structure biogenesis”, “Amino acid transport metabolism”, and “Nucleotide transport metabolism” categories (Hypergeometric test *p*-value < 0.05). (C) Bar plots depict the relative activity of RpoS and RpoH iModulons under heat shock conditions (mid-exponential phase at 42°C). The asterisk indicates the control conditions (mid-exponential phase at 37°C).

To investigate the roles of sigma factors in the heat shock response TRN, sigma factor binding profiles and gene expression profiles under both control and heat shock conditions were integrated (Fig 1A). A total of 1,353 differentially expressed genes (DEGs) (absolute log_2_ fold change ≥ 1.0 and a false discovery rate < 0.05) were observed by comparing the gene expression levels of *S*. Typhimurium before and after heat shock, with 713 genes up-regulated and 640 genes down-regulated (S1 Table). Functional analysis using clusters of orthologous groups (COG) categories was conducted to reveal functional enrichment in the heat shock response (Fig 1B). It was found that the majority of DEGs up-regulated by heat shock were enriched in intracellular trafficking/secretion vesicular transport (U), energy production conversion (C), and carbohydrate transport metabolism (G) (S1 Table). Additionally, COG categories related to cell wall/membrane/envelope biogenesis (M), cell motility (N), translation/ribosomal structure biogenesis (J), amino acid transport metabolism (E), and nucleotide transport metabolism (F) were statistically overrepresented among down-regulated genes.

To gain further insight into the transcriptional impact of heat shock, the heat shock response was analyzed by inferring the relative activity of iModulons using a strain-specific RNA-seq compendium (Fig 1A) [33]. iModulon activity, representing the relative changes in the expression of gene sets included in each iModulon compared to control conditions, was assessed (S1 Fig). The top 20 iModulons with the most notable changes in activity due to heat shock were identified, with 8 showing increased activity and 12 showing decreased activity (S1 Fig and Table 1). Among these, increased activity was observed in iModulons related to RpoD-family sigma factors (RpoS and RpoH) (Fig 1C), as well as in those associated with ppGpp (guanosine tetraphosphate). ppGpp, an alarmone produced under stress conditions, regulates various metabolic processes and interact with RNAP [34]. This suggests that regulation in *S*. Typhimurium is altered in response to heat shock by transitioning from RpoD to alternative sigma factors. Additionally, increased activity was also found in iModulons associated with *Salmonella* pathogenicity island-2 (SPI2, SPI2-T3SS), minor sigma factor (FliA-2), and transcription factors involved in carbon metabolism (Cra) and zinc metabolism (Zur). Conversely, decreased activity was observed in iModulons associated with anaerobic stress (Fnr-1), carbon metabolism (Mlc, MalT, GlpR), iron metabolism (Fur), amino acid metabolism (ArgR), nucleotide metabolism (PurR, PyrB-PyrI), flagellar biosynthesis (FlhD;FlhC), and autoinducer-2 regulation (LsrR) (S1 Fig and Table 1).

**Table 1:** Activity of the top 20 iModulons affected by heat shock.

iModulons	Control_1	Control_2	Heat shock_1	Heat shock_2	Attribution
**RpoS**	0.30	-0.30	34.45	34.82	Sigma factor
**ppGpp**	-0.10	0.10	14.58	15.21	Stress response
**SPI2-T3SS**	0.20	-0.20	7.15	6.91	Virulence
**SPI2**	-0.06	0.06	5.52	6.67	Virulence
**FliA-2**	0.00	0.00	5.28	5.45	Sigma factor
**Cra**	0.23	-0.23	5.22	5.32	Carbon metabolism
**Zur**	-0.22	0.22	5.13	5.55	Metal metabolism
**RpoH**	-0.04	0.04	5.12	5.10	Sigma factor
**Fur**	-0.28	0.28	-4.58	-5.42	Metal metabolism
**PyrB-PyrI**	0.01	-0.01	-4.72	-4.88	Nucleotide metabolism
**MalT**	0.29	-0.29	-5.38	-5.61	Carbon metabolism
**LsrR**	0.21	-0.21	-5.55	-5.54	Autoinducer-2 regulation
**GlpR**	-0.10	0.10	-5.80	-5.71	Carbon metabolism
**ArgR**	-0.24	0.24	-6.31	-6.18	Amino acid metabolism
**Mlc**	-0.05	0.05	-7.42	-6.76	Carbon metabolism
**Uncharacterized-6**	-0.29	0.29	-9.20	-6.16	Uncharacterized
**PurR**	-0.13	0.13	-9.94	-10.51	Nucleotide metabolism
**Fnr-1**	0.03	-0.03	-13.83	-13.54	Anaerobic stress
**FlhD;FlhC**	-0.17	0.17	-14.88	-15.26	Flagellar synthesis
**Uncharacterized-7**	0.36	-0.36	-17.57	-18.04	Uncharacterized

These findings highlight the complex and multifaceted impact of heat shock on transcriptional regulation across diverse metabolic processes in *S*. Typhimurium, providing strong guidelines for reconstructing heat shock response TRNs.

### Genome-wide binding profiles of sigma factors before and after heat shock treatment

**Fig 2:**
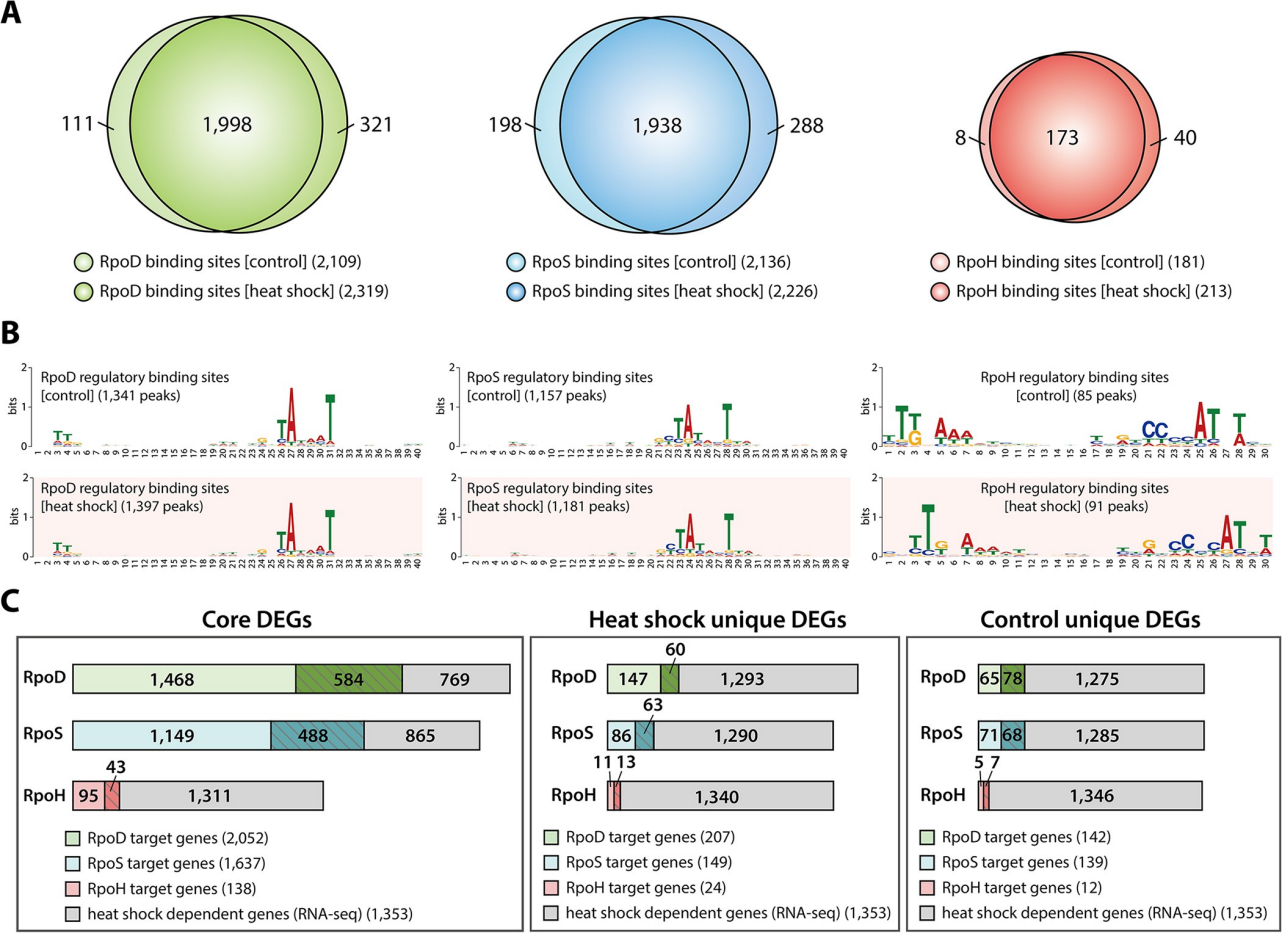
Changes in the heat shock TRNs regulated by RpoD-family sigma factors. (A) Venn diagrams show overlap of binding sites for each sigma factor before and after heat shock treatment. (B) Motif analysis of RpoS, RpoD and RpoH binding sites identified by ChIP-mini under both conditions. (C) Comparison of sigma factor binding results and differentially expressed genes (DEGs) to define sigmulon genes. Core DEGs represent heat shock DEGs found in the target genes with sigma factor binding under both control and heat shock conditions. Unique DEGs are those observed in target genes with sigma factor binding occurring exclusively under either control or heat shock conditions. Genes with expression changes with an absolute value of log_2_ fold change ≥ 1.0 and a false discovery rate < 0.05 are defined as differentially expressed.

To elucidate transcriptional regulation under heat stress by RpoD-family sigma factors, *in vivo* genome-wide binding profiles were generated using ChIP-mini method under control (37°C, Con) and sublethal heat shock (42°C, HS) conditions. Under control conditions, a total of 2,109, 2,136, and 181 binding sites were identified for RpoD, RpoS, and RpoH, respectively (Fig 2A and S2 Table). Following heat shock treatment, the emergence of new binding sites was confirmed, with counts of 321 for RpoD, 288 for RpoS, and 40 for RpoH (Fig 2A). Concurrently, a reduction in binding sites was observed, with losses amounting to 111, 198, and 8 for RpoD, RpoS, and RpoH, respectively (Fig 2A).

Given that RpoS and RpoH are members of the RpoD-family sigma factors, the overlap of binding sites was analyzed to investigate interactions between sigma factors. Over 41% of RpoS binding sites extensively overlapped with RpoD binding sites under both conditions (889/2,136: Con; 937/2,226: HS) (S2A Fig), indicating that RpoS binds to promoter sequences similar to those recognized by RpoD to coordinate transcriptional expression. Additionally, RpoH binding sites overlapped with those of RpoD by 28.7% (53/182) before and by 24.8% (54/214) after heat shock treatment, respectively (S2A Fig). Although the proportion of overlapped binding sites remained similar, the number of binding sites exclusively bound by RpoH increased, with the majority of these binding sites located in intragenic regions.

Previous studies have demonstrated that sigma factors bind to specific cis-acting sequence elements in the promoter region to regulate transcriptional initiation. To explore this further, the binding sites were categorized into regulatory and non-regulatory regions. The internal value of binding intensity within a single dataset was then calculated using signal-to-noise ratios, followed by sequence analysis (S2B Fig) [35–37]. As anticipated, the three sigma factors exhibited have significantly strong binding intensities in regulatory regions, indicating that these sigma factors predominantly bind to regulatory region, highlighting their crucial roles in transcriptional regulation (S2C Fig, Rank sum test *p*-value < 0.05). Additionally, the sequence motifs from the regulatory binding sites of these sigma factors were found to be consistent with those identified in previous studies (Fig 2B) [5,37].

To determine the causal relationship between sigma factors binding and changes in gene expression levels in response to heat shock, we defined the sigmulons for three sigma factors and compared them with DEGs induced by heat shock. Based on the ChIP-mini datasets, sigmulons for each sigma factor were identified under control (RpoD: 2,195 genes in 1,398 Transcription units (TUs); RpoS: 1,776 genes in 1,180 TUs; RpoH: 151 genes in 93 TUs) and heat shock conditions (RpoD: 2,259 genes in 1,446 TUs; RpoS: 1,786 genes in 1,191 TUs; RpoH: 163 genes in 98 TUs). In each sigmulon, genes present under both control and heat shock conditions and were significantly affected by heat shock were identified, revealing substantial changes in 584 RpoD, 488 RpoS, and 43 RpoH sigmulon genes (Fig 2C and S3 Table). Additionally, sigmulon genes that were exclusively present under heat shock conditions (RpoD: 60; RpoS: 63; RpoH: 13) or control conditions (RpoD: 78; RpoS: 68; RpoH: 7) and exhibited significant changes in expression levels were also identified (Fig 2C).

Consistent with previous studies suggesting that these sigma factors primarily act as positive effectors [5,24], sigmulon genes exclusively found under heat shock conditions were predominantly up-regulated, whereas those found only under control conditions were down-regulated (S2D Fig). Thus, the genome-wide measurement of binding sites for the three RpoD-family sigma factors, along with expression profiles, provides a precise investigation of the molecular interactions between sigma factors and promoters under heat shock conditions.

### Genome-wide identification of the RpoS sigmulon and its relationships with RpoD

Due to the limited pool of RNAP in a growing bacterial cell, each sigma factor must compete to associate with RNAP to initiate transcription [38]. RpoS is activated by various stress conditions, leading to increased binding at promoter regions to either directly initiate transcription or compete with RpoD [38,39]. As previously mentioned, a significant overlap in binding sites between RpoD and RpoS was observed, suggesting potential competition between these sigma factors at promoter regions.

**Fig 3:**
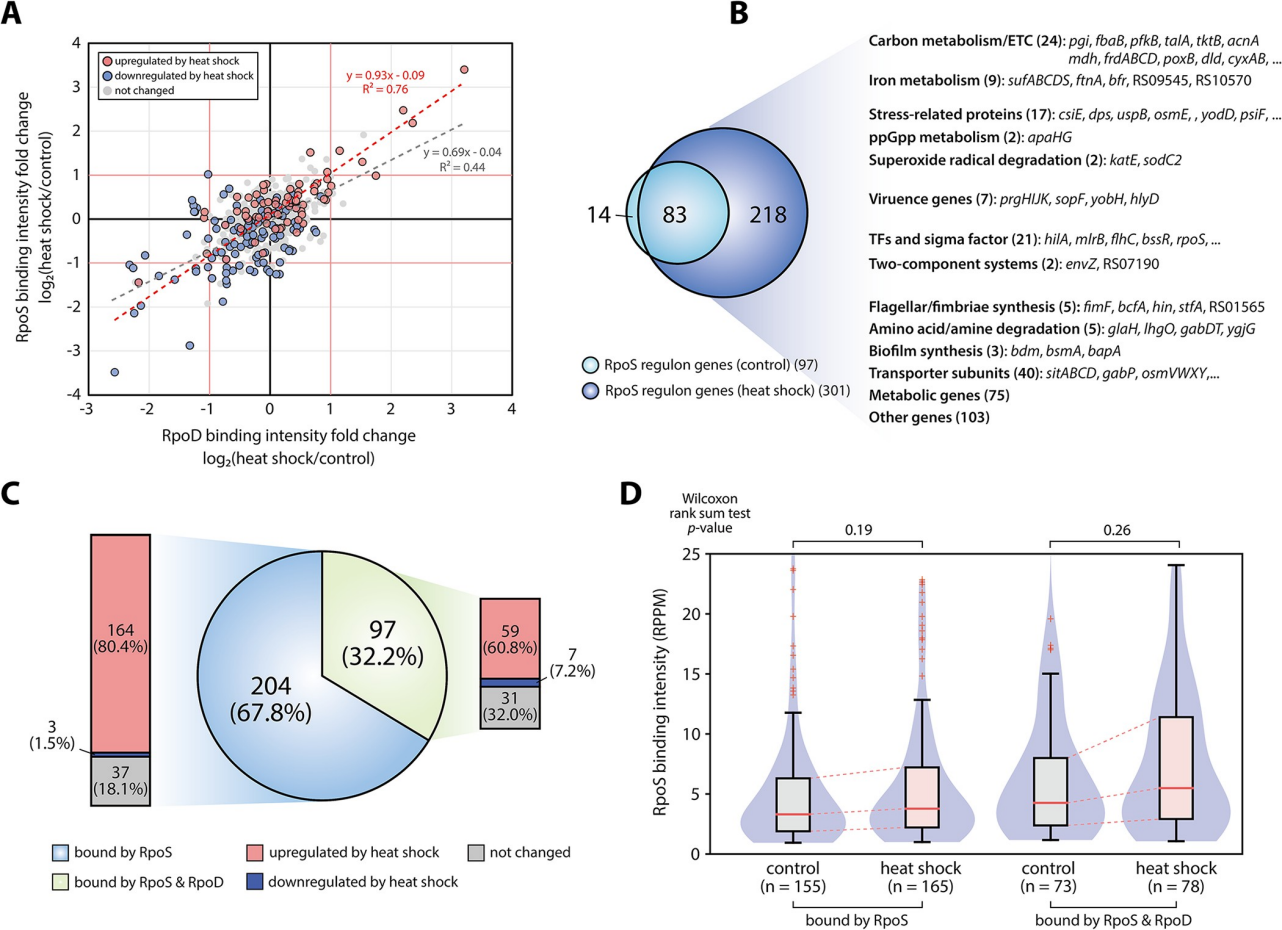
**Genome-wide identification of RpoS sigmulons in response to heat shock** (A) The scatter plot illustrates the fold changes in intensity of overlapping binding sites between RpoD and RpoS in response to heat shock. The grey dotted line indicates trend line for all overlapping binding sites, while the red dotted line denotes the trend line for overlapping binding sites with significant changes in binding intensity (absolute value of log_2_ fold change ≥ 1.0 and false discovery rate < 0.05). Red and blue circles represent up-regulated and down-regulated DEGs in response to heat shock, respectively. (B) Comparison of RpoS sigmulons between control and heat shock conditions. In response to heat shock, the number of RpoS sigmulon genes increased approximately threefold. The majority of sigmulon genes found under control conditions overlap with those under heat shock conditions. Under heat shock, RpoS regulates a total of 301 genes, including carbon metabolism and electron transport chain (24 genes), iron metabolism (9 genes), stress-related proteins (16 genes), ppGpp metabolism (2 genes), superoxide radicals degradation (2 genes), virulence (3 genes), transcription factors and sigma factors (21 genes), two-component systems (2 genes), flagellar and fimbriae synthesis (4 genes), amino acid/amine degradation (5 genes), biofilm synthesis (3 genes), transporter subunits (39 genes), metabolic processes (72 genes), and other functions (99 genes). (C) Overlap of target genes for RpoD and RpoS within RpoS sigmulon genes under heat shock conditions. (D) Changes in binding intensity within RpoS sigmulons before and after heat shock. The binding intensity of RpoS at promoter regions was similar between control and heat shock conditions when bound only by RpoS. In contrast, the binding intensity of sites bound by RpoD and RpoS increased in response to heat shock.

To investigate changes in sigma factor binding intensity before and after heat shock, a comparative analysis was conducted, normalizing the binding intensity into RPPM values using the DiffExo pipeline (ChIP-exo peak normalization pipeline) [31]. A total of 162 and 127 binding sites for RpoD and RpoS, respectively, were identified as differentially binding peaks (DBPs) (absolute value of log_2_ intensity fold change ≥ 1.0 and false discovery rate < 0.05). Notably, 46 of the 127 RpoS DBPs overlapped with RpoD DBPs (Fig 3A). The change in binding intensity of the total overlapping sites (r^2^ = 0.44) exhibited a similar pattern between RpoD and RpoS, with an even more pronounced similarity observed in DBPs (r^2^ = 0.76) (Fig 3A). Furthermore, the binding intensity of both sigma factors correlated with the expression levels of their target genes, underscoring their primary function as positive effectors (S3A Fig). These similarities in binding intensity changes between RpoD and RpoS highlight the competitive dynamics of sigma factors at promoter regions, particularly within RpoS sigmulon, which include genes directly regulated by RpoS under heat shock conditions. This raises important questions about the detailed molecular mechanisms underlying sigma factor competition in response to stress.

To identify RpoS sigmulons, the transcript expression levels of RpoS-regulated genes were compared between the wild-type and the *rpoS* deletion mutant (*ΔrpoS*). A total of 97 sigmulon genes were differentially expressed under control conditions, while 301 sigmulon genes were differentially expressed under heat shock conditions (Fig 3B and S4 Table). This analysis revealed that RpoS acts primarily as a positive effector but also exerts a negative effect on the transcriptional expression of some genes (S3B Fig). The heat shock treatment significantly expanded the size of the RpoS sigmulons, consistent with the observed increase in RpoS iModulon activity (Fig 1C). These RpoS sigmulons include numerous genes related to various metabolic and cellular processes (Fig 3B). Notably, the RpoS sigmulons encompass genes involved in carbon metabolism, iron metabolism, and the electron transport chain (ETC), as well as stress response genes related to stress-related proteins, ppGpp metabolism, and superoxide radical degradation (Fig 3B). In addition, RpoS exhibits a distinctive regulatory mechanism by not only directly activating virulence proteins (*sopF*, *yobH*, *hlyD*) but also repressing genes associated with the SPI-1 type III secretion system (T3SS) (*prgHIJK*) and SPI-1 regulator (*hilA*) (S3B Fig).

Out of 301 RpoS sigmulon genes, 97 genes (32.2%) have both RpoD and RpoS binding sites upstream (Fig 3C). Approximately 80.4% of the genes bound only by RpoS showed increased expression levels in response to heat shock, while only 60.8% of the genes bound by both RpoD and RpoS were up-regulated (Fig 3C). This suggests that RpoD competes with the abundant RpoS induced by heat shock, thereby limiting RpoS access to the promoter region. Consistent with these observations, the intensity of RpoS-specific binding sites showed a slight increase, whereas binding sites associated with shared promoters exhibited a more pronounced enhancement (Fig 3D). Furthermore, at shared promoters, the binding intensity for RpoD was significantly lower than for RpoS (T-test *p*-value < 0.05), and the binding intensity of RpoD did not notably increase upon heat shock. (S3C Fig). These indicate that RpoS especially increases its binding within promoter regions shared with RpoD under heat shock conditions.

To further explore the biological significance of this competition, we identified 30 RpoS-regulated genes associated with 22 overlapping binding sites, which exhibited more than a 1.5-fold increase in binding intensity in response to heat shock (S4 Table). In carbon metabolism, RpoS binding upstream of *mdh*, *galT*, and *glk* increased, while the expression levels of these genes did not change significantly. This result suggests that RpoD may continue to play a dominant role in regulating carbon metabolism following heat treatment. Despite the dominance of RpoD, 17 out of 30 genes showed significant up-regulation due to increased RpoS binding in response to heat shock. Notably, the virulence-related transcription factor *mlrB* was identified among the up-regulated genes. MlrB is known as a SPI-2-encoded *Salmonella*-specific transcription factor that represses the biofilm formation regulator *csgD* under intra-macrophage conditions [40]. However, after heat treatment, a significant increase in *csgD* expression was observed, suggesting the possibility of alternative functions of MlrB activated by RpoS under extra-macrophage conditions. Moreover, RpoS strongly activated gene expression by increasing its binding upstream of *ubiCA* (ubiquinol-8 biosynthesis), *bdm* (biofilm formation), and STM14_RS09545 (iron metabolism) through competition with RpoD. These findings expand our understanding of the competition mechanisms between RpoS and RpoD in various metabolisms.

### Transcriptional regulation of heat shock proteins by RpoH

**Fig 4:**
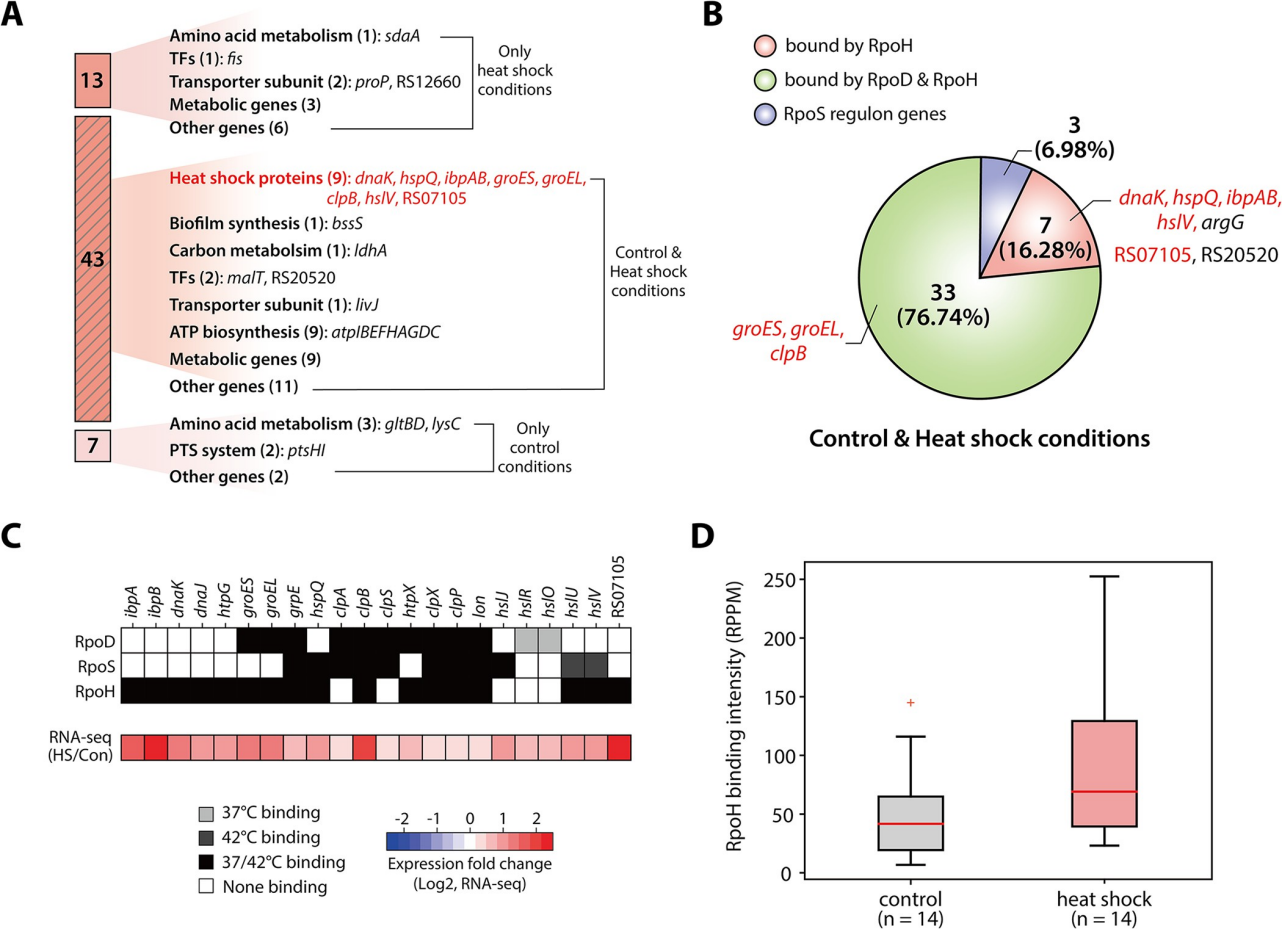
**Transcriptional regulation of heat shock-related protein genes by RpoH during heat shock conditions** (A) Comparison of RpoH target DEGs between control and heat shock conditions. (B) Overlap of RpoH target genes with those of other sigma factors, illustrating the intersection of RpoH-bound genes with RpoD and RpoS sigmulons under both control and heat shock conditions. (C) The mRNA expression levels of heat shock-related protein genes were assessed before and heat shock treatment. Genes significantly upregulated in response to heat shock are indicated with three asterisks (log_2_ fold change ≥ 1.0 and false positive rate <0.001). (D) Heat map showing sigma factor binding and expression changes for heat shock-related protein genes in response to heat shock. The upper three stacks denote the binding of each sigma factor (RpoD, RpoS, and RpoH), and the bottom stack indicates the relative expression changes of these genes between heat shock (42°C) and control conditions (37°C). (E) Box plot illustrates the intensity of RpoH binding observed upstream of heat shock-related protein genes under control and heat shock conditions, showing increased binding intensity in response to heat shock.

Heat shock proteins (HSPs) are essential components of the cellular machinery that protect cells against elevated temperatures. These proteins, including molecular chaperones and proteases, stabilize other proteins by ensuring proper folding and degrading misfolded proteins. Our analysis identified nine HSP genes (*dnaK*, *hspQ*, *ibpAB*, *hslV*, *groES*, *groEL*, *clpB*, and STM14_RS07105) as being bound by RpoH under both control and heat shock conditions, exhibiting significantly increased expression levels in response to heat shock (Fig 4A). Among these, six genes were exclusively bound by RpoH, and three genes shared binding sites with RpoD (Fig 4B).

To further explore the genome-wide binding profiles and expression changes of HSP genes, 22 HSP genes with at least one sigma factor binding site were identified (Fig 4C). None of these HSP genes were directly regulated by RpoS. Additionally, five HSP genes (*clpA*, *clpS*, *hslJ*, and *hslRO*) did not have RpoH binding at their promoter regions and did not show significant changes in expression upon heat shock, except for *hslJ*. The *hslJ* gene exhibited a considerably low expression level under control conditions, making it more sensitive to expression changes (S4 Fig). To identify relationships between RpoH binding intensity and gene expression levels, changes in binding intensity upstream of significantly upregulated HSP genes were analyzed (Fig 4D). The results revealed that binding intensity increased after heat shock treatment. Thus, these findings indicate that HSP genes of *S*. Typhimurium are primarily regulated by increased RpoH binding at promoter regions in response to heat shock.

### Regulation of electron transport chain and carbon metabolism genes by indirect anaerobic stress due to heat shock

**Fig 5:**
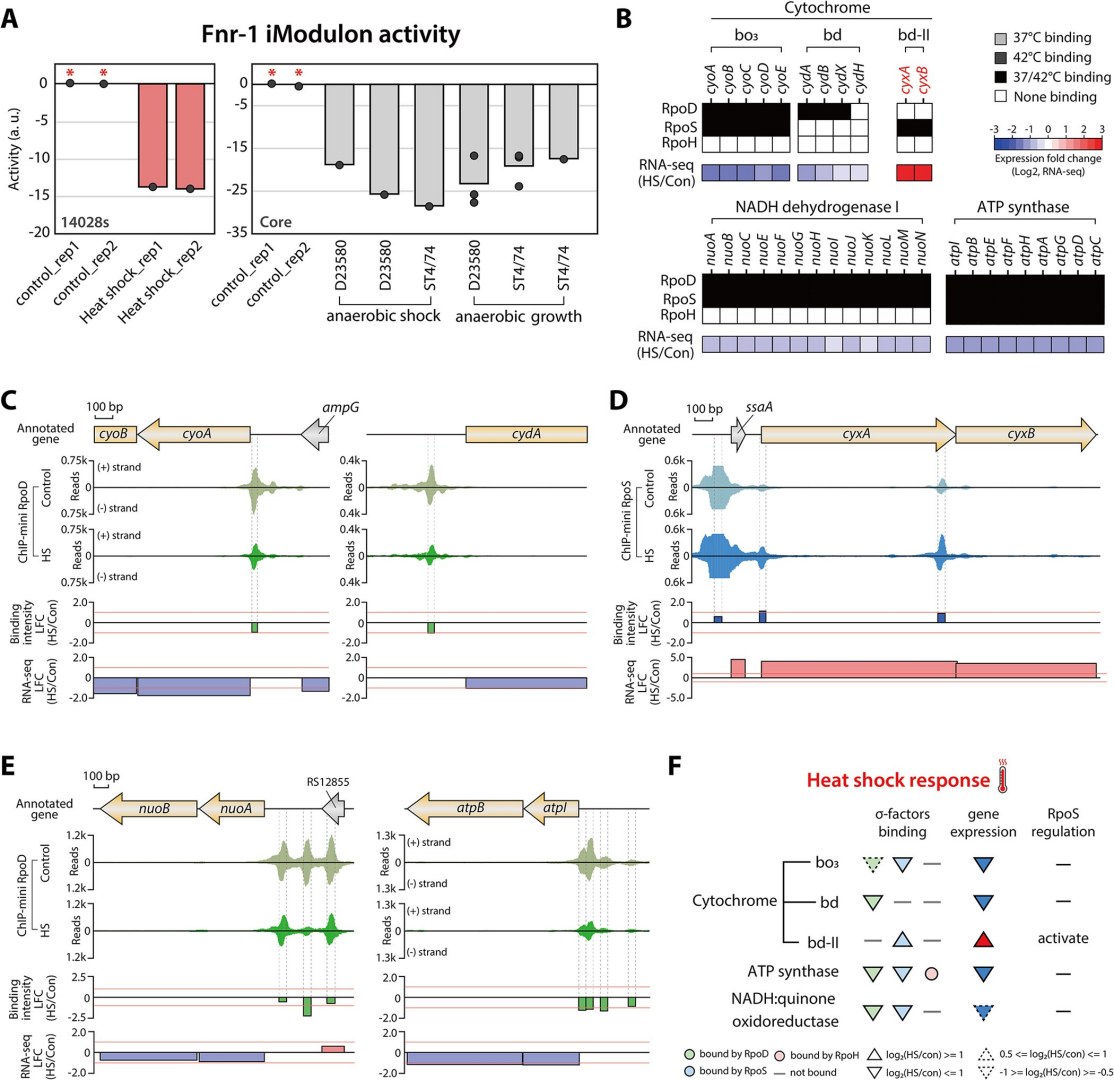
**Transcriptional regulation of electron transport chain genes in response to heat shock, mirroring anaerobic stress** (A) Bar plots depicting the relative activity of the Fnr-1 iModulon under heat shock conditions (mid-exponential phase at 42°C). Red bars denote the activity of Fnr-1 in response to heat shock, inferred using the *S*. Typhimurium 14028s RNA-seq compendium. Grey bars illustrate the activity of Fnr-1 in response to anaerobic shock and anaerobic growth in the *S*. Typhimurium core RNA-seq compendium. The asterisk indicates the control conditions (mid-exponential phase at 37°C). (B) Heat map of sigma factor binding and expression change for electron transport chain genes in response to heat shock. Expression levels of cytochrome bo_3_, cytochrome bd, ATP synthase, and NADH dehydrogenase I genes are down-regulated by heat shock, while cytochrome bd-II genes (*cyxAB*) are significantly up-regulated by RpoS under heat shock conditions. The upper three stacks denote the binding of each sigma factor, and the bottom stack indicates relative gene expression changes between heat shock (42°C) and control conditions (37°C). In addition, genes up-regulated by RpoS are shown in red, and those down-regulated by RpoS are represented in blue. (C) RpoD binding events upstream of TUs for cytochrome bo_3_, cytochrome bd. The binding intensity of RpoD upstream of *cyoABCDE* and *cydAB* decreases in response to heat shock. (D) RpoS binding events upstream of *ssaA*-*cyxAB* transcription units (TUs). The binding intensity of RpoS upstream of *cyxAB* significantly increases in response to heat shock. (E) RpoD binding events upstream of TUs for NADH:quinone oxidoreductase and ATP synthase. Among the three RpoD binding sites upstream of *cyoABCDE*, only the binding intensity at the site corresponding to the transcription start site (TSS) significantly decreases. Additionally, a significant reduction in RpoD binding is also observed for *atpIBEFHAGDC*. (E) Differential transcriptional regulation of electron transport chain genes by sigma factors in response to heat shock. This schematic summarizes how heat shock impacts sigma factor binding and gene expression, highlighting the regulatory roles of RpoS and RpoD in modulating the expression of electron transport chain components.

Fnr is a primary transcription factor known to regulate various metabolic and ETC genes in response to low oxygen levels [41,42]. Based on the iModulon analysis of the *Salmonella* core RNA-seq compendium, which compiles 534 *Salmonella* gene expression profiles [33], decreased Fnr-1 activity was observed under anaerobic shock and anaerobic growth conditions (Fig 5A). This suggests that the expression levels of gene sets within the Fnr-1 iModulon are down-regulated by anaerobic stress. Similarly, the relative activity of Fnr-1 was reduced following heat shock treatment (Fig 5A), indicating that anaerobic stress may also be implicated in the heat stress.

First, to investigate the effect of heat shock on the ETC, changes in sigma factor binding and expression of ETC genes were analyzed under both control and heat shock conditions (Fig 5B). Expression levels of cytochrome oxidases (bo_3_ and bd), NADH dehydrogenase, and ATP synthase genes were reduced, with *cyoABCDE*, *cydA*, and *atpIBEFHAGDC* being especially significantly down-regulated by heat shock. Interestingly, cytochrome bd-II oxidase (*cyxAB*) showed notable increases in expression level, which were directly up-regulated by RpoS (Fig 5B). The *cyxA* gene is known to provide a fitness advantage during growth at 0.8% oxygen, indicating a response to lower oxygen levels [43,44]. The increased expression of *Salmonella cyxAB* in response to heat shock suggests that heat shock may be accompanied by anaerobic stress.

To further explore the relationship between changes in sigma factor binding intensity and expression levels in response to heat shock, sigma factor binding sites upstream of ETC genes were identified. As anticipated, the binding intensity of RpoD at *cyoABCDE* and *cydAB* decreased, suggesting that reduced RpoD binding impacts RNAP recruitment, leading to down-regulation of expression (Fig 5C). In contrast, RpoS binding at the promoter region of *cyxAB* exhibited a significant increase, correlating with elevated gene expression levels (Fig 5D). Additionally, the binding intensity of RpoD upstream of NADH dehydrogenase and ATP synthase genes also decreased (Fig 5E). Specifically, among the RpoD binding sites of NADH dehydrogenase genes, those corresponding to transcription start site (TSS) regions were more significantly impacted by heat shock (S5 Fig). Thus, heat shock triggers a reduction in RpoD and RpoS binding upstream of major ETC genes, resulting in down-regulation of their expression levels (Fig 5F). Additionally, the notable increase in RpoS binding and expression levels in *cyxAB* may indicate an adaptive mechanism to respond to heat-induced anaerobic stress (Fig 5B). Thus, these results underscore that heat shock not only imposes thermal stress but also anaerobic stress, affecting the transcriptional regulation for the ETC in *S*. Typhimurium.

**Fig 6:**
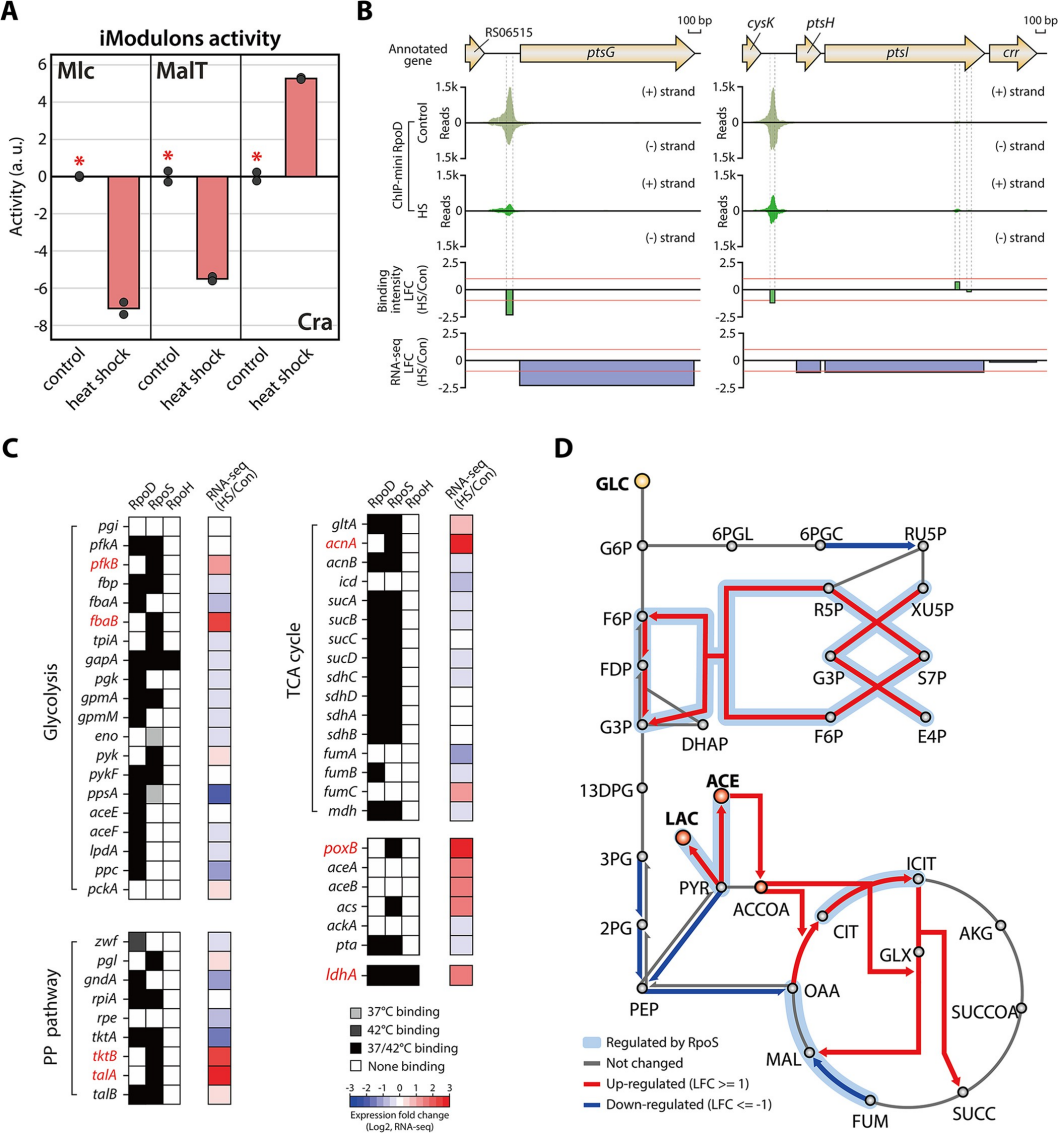
**Heat shock induces fluctuations in carbon metabolism in *Salmonella*** (A) Bar plot depicting the relative activity of iModulons related to carbon metabolism including Mlc, MalT, and Cra, under heat shock condition (mid-exponential phase at 42°C). Asterisk indicates the control condition (mid-exponential phase at 37°C). (B) RpoD binding events upstream of the phosphotransferase system (PTS) genes under four conditions. The binding intensity of RpoD upstream of *ptsG* and *ptsHI* significantly decreases in response to heat shock. (C) Heat map showing sigma factor binding and expression change for carbon metabolism genes in response to heat shock. RpoS directly up-regulates five isozymes involved in carbon metabolism including glycolysis, the pentose phosphate pathway, and the TCA cycle under heat shock conditions. Additionally, the expression of acetate and lactate metabolism genes is significantly up-regulated. The left two stacks denote the binding of RpoD and RpoS, and the rightmost stack indicates the relative expression of genes between heat shock (42°C) and control conditions (37°C). Genes up-regulated by RpoS are shown in red, and those down-regulated by RpoS are represented in blue. (D) Diagram showing the overall differentially expressed genes involved in carbon metabolism in response to heat shock. Metabolic pathways regulated by RpoS sigmulon genes are highlighted with a bold light blue line.

Since the ETC and carbon metabolism are closely interconnected through electron donors derived from carbon metabolism pathway, significant down-regulation of ETC genes was expected to impact carbon metabolism. Based on iModulon analysis, three iModulons (Mlc, MalT, and Cra) showed altered activity due to heat shock (Fig 6A). Mlc is a global regulator that represses various genes and operons involved in glucose uptake and metabolism [45,46]. It also represses the maltose system transcription activator, *malT* [46]. Therefore, the decreased iModulon activity of Mlc and MalT suggests down-regulation of gene sets within their respective regulons. This may indicate that the repressor Mlc is activated by heat shock, leading to the repression of glucose uptake and metabolism. The reduction in MalT iModulon activity further supports activation of Mlc, which represses *malT* (Fig 6B).

To determine how heat shock impacts the transcription of glucose uptake genes, changes in RpoD binding intensity and expression levels of PTS system genes (*ptsG*, *ptsHI*-*crr*) were examined (Fig 6B). As expected, the intensity of two RpoD binding sites upstream of *ptsG* and *ptsHI*-*crr* showed significant reduction, and their expression levels were also down-regulated by heat shock. Furthermore, alterations in sigma factors binding and expression levels of core carbon metabolism were also analyzed (Fig 6C). While no significant differences were found between control and heat shock conditions, indicating overall weak and partial strong down-regulation of carbon metabolism genes, an interesting property emerged in the regulation of isoenzymes. Under heat shock conditions, expression of RpoD-regulated isoenzymes (*pfkA*, *fbaA*, *tktA*, *talB*, and *acnB*) was down-regulated or unchanged, whereas isoenzymes within the RpoS sigmulon (*pfkB*, *fbaB*, *tktB*, *talA*, and *acnA*) were significantly up-regulated (Fig 6C). RpoS also activated pyruvate oxidase (*poxB*) and lactate dehydrogenase (*ldhA*), which convert pyruvate to acetate and lactate, respectively (Fig 6C). Moreover, partial TCA cycle and glyoxylate shunt genes for acetate metabolism (*aceAB*) were substantially up-regulated (Fig 6D). This corresponds to increased iModulon activity of Cra, a transcription factor regulating carbon metabolism, particularly for poor carbon sources such as acetate (Fig 6A) [47]. These results suggest that heat shock not only down-regulates the expression of ETC genes but may also induce the conversion of pyruvate to acetate, potentially decreasing carbon flux through the TCA cycle. This is consistent with previous study in *Escherichia coli*, which show that higher temperature increases acetate formation [48]. Additionally, the activation of Cra-associated regulons involved in acetate metabolism and the glyoxylate shunt may provide TCA intermediates under heat shock conditions.

Consequently, this transcriptional regulation of carbon metabolism, coupled with the reduction in ETC expression, suggests a deceleration of bacterial cell growth to prioritize the refolding and stabilization of proteins damaged by heat shock [39]. In this process, RpoS, along with ppGpp, a nucleotide alarmone produced by SpoT or RelA in response to stress, likely plays a crucial role in sustaining cell growth under heat shock conditions [49]. In *E*. *coli*, ppGpp activates the transcription of anti-adapter protein genes to increase RpoS stability, depending on the type of stress, such as *iraP* (phosphate limitation) and *iraD* (steady state) (S6A Fig) [50]. Moreover, *iraP* in *S*. Typhimurium is required for accumulation of the RpoS protein during phosphate or Mg^2+^-limiting conditions [51]. Gene expression profiles showed significant up-regulation of *iraP*, indicating that heat shock may induce ppGpp generation to increase RpoS stability via IraP, similar to phosphate or Mg^2+^-limiting conditions, an important virulence condition (S6B Fig). Additionally, increased ppGpp iModulon activity supports these findings (S1 Fig).

### *S*. Typhimurium regulates iron metabolism to mitigate iron-mediated oxidative stress

The toxicity of hydrogen peroxide, a by-product of oxidative metabolism in bacteria, primarily arises from DNA damage caused by the Fenton reaction, which generates reactive hydroxyl radicals from hydrogen peroxide using intracellular free iron [52–54]. This reaction is known to accelerate with an increase in temperature, underscoring the significance of iron metabolism in *S*. Typhimurium following heat shock treatment [55].

**Fig 7:**
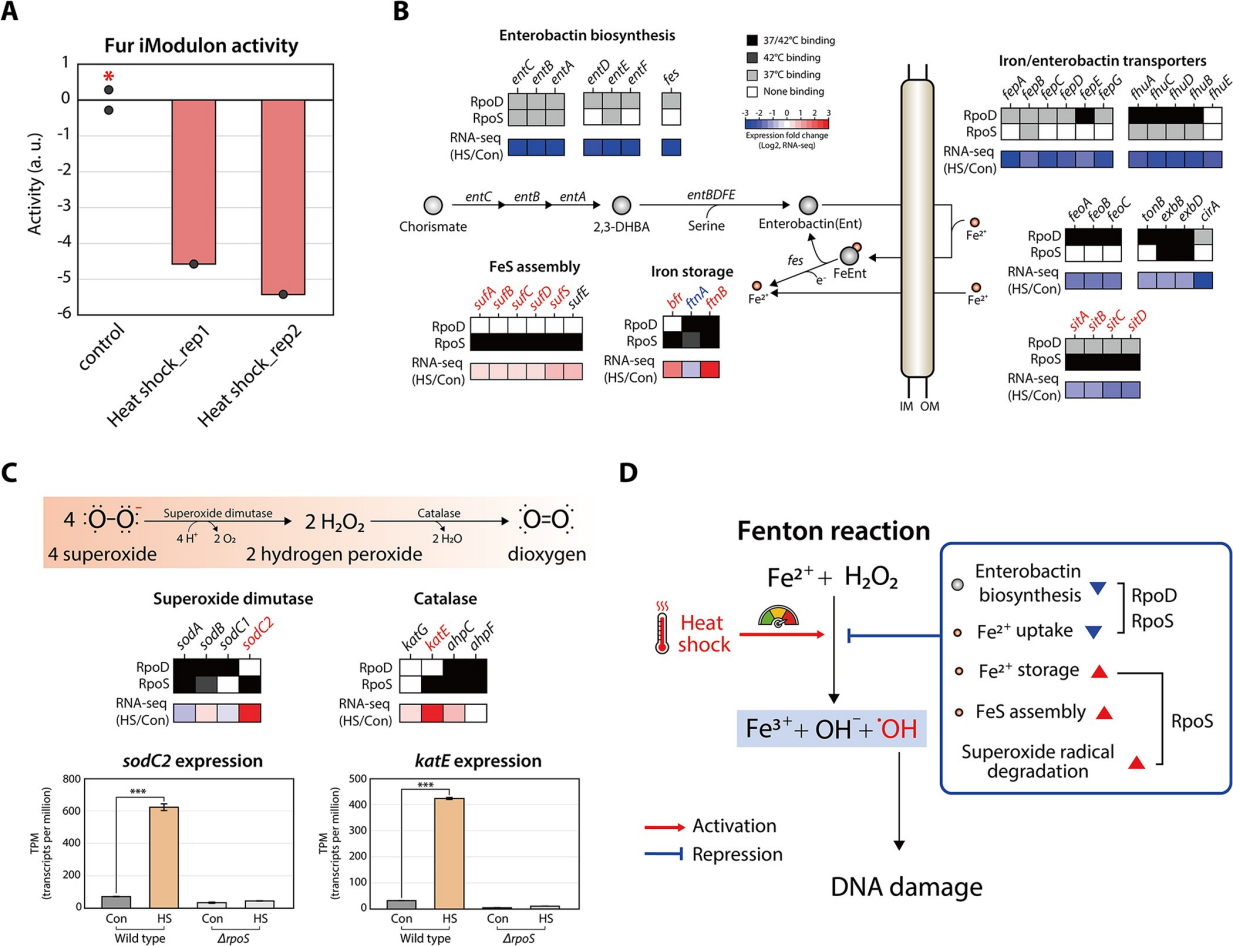
**Regulation of iron acquisition/storage and superoxide radical degradation pathways to mitigate Fenton reaction caused by heat shock** (A) Bar plot depicting the relative activity of the Fur iModulon under control conditions (mid-exponential phase at 37°C). The asterisk indicates the control conditions (mid-exponential phase at 37°C). (B) Representation of the iron acquisition (enterobactin biosynthesis and iron/enterobactin transport), iron storage, and FeS assembly pathways. Expression levels of iron acquisition genes are down-regulated, whereas intracellular iron storage and FeS assembly genes are up-regulated in response to heat shock, possibly leading to a reduction in intracellular iron concentration. The upper two stacks denote the binding of RpoD and RpoS, and the bottom stack indicates the relative gene expression changes between heat shock (42°C) and control conditions (37°C). Genes up-regulated by RpoS are shown in red, and those down-regulated by RpoS are represented in blue. (C) Illustration of the superoxide radical degradation pathway and corresponding mRNA expression levels in wild-type and *ΔrpoS* strains. (D) Schematic diagram illustrating the transcriptional regulation mechanisms that mitigate the Fenton reaction under heat shock conditions. Heat shock can accelerate the Fenton reaction, producing damaging hydroxyl radicals from hydrogen peroxide. This is mitigated by the down-regulation of iron acquisition and the up-regulation of iron storage and superoxide radical degradation, regulated by RpoS and RpoD. 2,3-DHBA: 2,3-dihydroxybenzoic acid, IM: inner membrane, OM: outer membrane.

Based on iModulon activity analysis, the activity of the Fur iModulon, a ferric uptake regulator controlling a variety of iron metabolism genes, was found to decrease in response to heat shock (Fig 7A). To investigate detailed fluctuations in iron metabolism under heat shock conditions, the binding of sigma factors and expression levels across four iron-related processes (enterobactin biosynthesis, iron/enterobactin transporters, FeS assembly, and iron storage) were identified (Fig 7B). Under heat shock conditions, RpoD binding to regions upstream of the entire enterobactin biosynthesis genes and part of the enterobactin/iron transporter subunit genes was abolished, resulting in significant down-regulation of their expression levels (Fig 7B). Additionally, five RpoD binding sites that overlapped between control and heat shock conditions exhibited reduced binding intensity, with notable decreases observed upstream of *fepE*, *fhuACDB*, *feoABC*, and *exbBD* (Table 2 and S7 Fig). Conversely, FeS assembly and iron storage genes, which utilize intracellular iron, exhibited increased expression levels (Fig 7B). Moreover, an interesting transcriptional regulatory role of RpoS in iron-related processes was observed. RpoS was found to activate *sitABCD*, *sufABCDS*, *bfr*, and *ftnB*, while repressing *ftnA* (Fig 7B). Among these findings, RpoS binding upstream of *sitABCD* showed reduced intensity accompanied by down-regulation of gene expression levels. In contrast, binding upstream of *sufABCDS* and *ftnB* slightly increased, correlating with up-regulation of their gene expression levels (Table 2 and S8 Fig). Additionally, RpoS binding upstream of *ftnA* was exclusively detected under heat shock conditions. Furthermore, transcriptional regulation by RpoS also contributes to alleviating oxidative stress by converting superoxide radicals into dioxygen. Among superoxide dismutase and catalase genes in *S*. Typhimurium, only *sodC2* and *katE* were found to be significantly up-regulated by RpoS, facilitating the removal of superoxide radicals under heat shock conditions (Fig 7C).

**Table 2:** Binding intensity change in iron metabolism genes.

	RpoD binding sites			RpoS binding sites		
	Start	End	LFC	Start	End	LFC
*fepE*	650,221	650,269	-1.36	N.D	N.D	-
*fhuACDB*	224,355	224,409	-1.81	224,397	224,444	-
*feoABC*	3,677,670	3,677,721	-1.79	N.D	N.D	-
*tonB*	1,842,682	1,842,733	-0.78	N.D	N.D	-
*exbBD*	3,338,993	3,339,044	-1.33	3,339,210	3,339,261	0.49
*sitABCD*	3,026,537	3,026,586	-	3,026,550	3,026,601	-1.09
*sufABCDSE*	N.D	N.D	-	1,462,167	1,462,216	0.69
*bfr*	N.D	N.D	-	3,611,692	3,611,743	-0.26
*ftnA*	2,040,739	2,040,788	0.38	2,040,737	2,040,786	-
*ftnB*	2,038,477	2,038,526	0.36	2,038,550	2,038,601	0.80

These results indicate that *S*. Typhimurium alters its TRN in response to heat shock by decreasing RpoD and RpoS binding upstream of enterobactin biosynthesis and iron/enterobactin uptake genes, while increasing RpoS binding upstream of FeS assembly and iron storage genes that utilize intracellular iron (Fig 7D). This strategy likely aims to down-regulate the expression levels of extracellular iron uptake genes and up-regulate genes involved in utilizing intracellular iron. Consequently, this reduces intracellular iron levels, thereby diminishing DNA damage caused by oxidative stress from the Fenton reaction. Additionally, RpoS not only regulates iron metabolism but also plays an important role in the degradation of superoxide radicals by significantly upregulating two genes, *sodC2* and *katE* (Fig 7C).

## Discussion

This study presents a comprehensive genome-wide reconstruction of the regulatory network of RpoD-family sigma factors in *S*. Typhimurium, utilizing machine learning-based transcriptome analysis to extend our understanding of their roles in heat shock adaptation beyond the expression of HSPs. We identified genome-wide binding maps of RpoD, RpoS, and RpoH with near-1bp resolution under both control and heat shock conditions using the ChIP-mini method. Additionally, heat shock-dependent and RpoS-dependent transcripts generated by RNA-seq were integrated with binding profiles, including quantitative fluctuations in binding intensity, to elucidate the direct or indirect transcriptional regulatory roles of each sigma factor.

From this analysis, it was revealed that RpoD-family sigma factors of *S*. Typhimurium sophistically change their intensity to dynamically regulate gene expression levels under heat shock conditions. Machine learning-based iModulon analysis was employed to decompose transcriptional impact of heat shock, revealing that heat stress is accompanied by anaerobic and oxidative stresses. By integrating these findings with genome-scale data, we identified crucial relationships between changes in RpoD and RpoS binding upstream of TSS and gene expression levels under heat shock conditions. These changes shift the transcriptional preferences of ETC and carbon metabolism genes in response to heat-induced anaerobic stress, while also regulating iron availability genes to mitigate oxidative stress caused by the Fenton reaction.

Notably, RpoD binding upstream of iron metabolism genes (*entD*, *fepA*, *fes*-RS03545-*enrF*, *fepCGD*, *entS*, *fepB*, *entCEBAH*, *cirA*), and iron transporter subunit genes (*sitABCD*) was abolished, along with down-regulation of their expression levels (Fig 7B). Incidentally, a divergent trend in relative activity of iModulon FliA-2 (12 genes) and FlhD; FlhC (41 genes) related to flagellar biosynthesis was observed. Considering the number of genes included in each iModulon, it is reasonable to infer that heat shock has an inhibitory effect on flagellar biosynthesis. These results were consistent with ChIP-mini data, which indicated the loss of RpoD binding upstream of flagellar biosynthesis genes (*flgA*, *flgBCDEFGHIJK*, *flhBA*, *fliDST*, *fliE*, *fliFGHIJK*). Moreover, we found that heat shock inhibits arginine and nucleotide biosynthesis at the transcriptional level. This transcriptional regulation mechanism was evidenced by the abolition of RpoD binding or significant reductions in the binding intensity of RpoD and RpoH (S9 Fig).

Previously, a study of *S*. Typhimurium in Luria Bertani media showed up-regulation of SPI-2 and SPI-5 genes, along with down-regulation of SPI-1-encoded genes in response to heat stress [4]. To assess the impact of heat stress on the expression levels of virulence-related genes in our datasets, transcriptional changes in six SPIs were identified (S10A Fig). These results showed that SPI-1 had the highest expression levels under both optimal temperature and heat shock conditions. Genes related to SPI-2 and SPI-6 exhibited significant up-regulation in response to heat shock; however, the overall expression levels of SPI genes were relatively low due to the sampling conditions were not optimal for pathogenic gene expression. Additionally, the activity of SPI-2 and SPI-2-T3SS iModulons also increased (S10B Fig), but the majority of sigma factor binding upstream of TSS regions was not detected under either condition (S10C Fig). Therefore, further heat shock studies of *S*. Typhimurium under various infection-relevant conditions using our workflow will offer an opportunity to deepen our understanding of the pivotal roles RpoD-family sigma factors play in pathogenicity.

Our systems biology approach has comprehensively elucidated the complex roles of the regulatory network involving three representative RpoD-family sigma factors in *S*. Typhimurium in response to heat shock. First, we identified a competition mechanism between RpoS and RpoD, where RpoS increases its binding at overlapping sites upstream of the promoter region to compete with RpoD under heat shock conditions. Additionally, we observed an increase in RpoH binding upstream of genes encoding HSPs, chaperons, and proteases. Second, heat-induced anaerobic stress down-regulated the majority of ETC genes, leading to changes in the TRN of carbon metabolism. This shift could also be influenced by ppGpp, which is produced under stressful conditions and co-regulates the glyoxylate shunt (*aceAB*) and acetate metabolic genes (*poxB*) in *E*. *coli* [56,57]. However, unlike in *E*. *coli*, RpoS binding upstream of *aceAB* was not observed in *S*. Typhimurium, suggesting that the expression of these genes may be governed by different transcriptional regulatory mechanisms, potentially involving other sigma factors (S11 Fig). Interestingly, under heat shock conditions, RpoS directly up-regulated cytochrome bd-II oxidase (*cyxAB*), as well as isoenzymes involved in carbon metabolism, indicating that RpoS plays a more important role than RpoH in the transcriptional regulation of metabolic processes for adapting to sublethal heat stress. Finally, we found that changes in iron availability led to the downregulation of extracellular iron uptake transporters and enterobactin biosynthesis/transporters, while intracellular iron-utilizing genes were upregulated. This regulation might help reduce intracellular iron levels to mitigate oxidative stress caused by the Fenton reaction.

To assess whether our findings are exclusive to *S*. Typhimurium, we conducted a comparative analysis using a recent study on the heat shock response in other bacteria. We compared the heat shock response in *E*. *coli*, which is closely related to *S*. Typhimurium [48]. Similar to our findings, heat shock induced down-regulation of PTS system and ETC genes in *E*. *coli*, leading to acetate formation due to decreased TCA cycle activity [48]. However, detail transcription expression of genes showed different preference between both species. In contrast to *S*. Typhimurium, heat shock induces the activation of *ackA* while repressing *acs*, the acetate transporter gene, in *E*. *coli* (Fig 6C). Additionally, *E*. *coli* possesses a single operon of cytochrome bd-II (*appCB*-*appX*), whereas *S*. Typhimurium has two homologs (*appCB*-*appX* and *cyxAB*). Under heat shock conditions, *E*. *coli* was found to activate cytochrome bd (*cydB*) rather than cytochrome bd-II (*appCB-appX*), suggesting that *cyxAB* in *S*. Typhimurium plays a distinct role in adaptation to heat stress. However, precise physiological role of this unique homolog (*cyxAB*) in heat shock is still unclear, highlighting the need for further investigation.

Furthermore, we compared our findings to those of bacteria within host environments to identify differences in the heat shock response under intracellular conditions. *Chlamydia trachomatis* is an obligate intracellular bacterium that causes sexually transmitted infections [58,59]. Interestingly, heat shock in intracellular *C*. *trachomatis* triggers the activation of energy production genes to generate NADH, which is used in oxidative phosphorylation [59]. These results differ from our findings of repressed oxidative phosphorylation gene expression and activated acetate metabolism (Figs 5B and 6C). This suggests that *C*. *trachomatis* may flexibly adjust its heat shock response TRNs to intracellular conditions, as it utilizes 2-oxoglutarate instead of glucose as a carbon source inside the host. Thus, this suggests that there may be differences in *Salmonella*’s heat shock response between intracellular and extracellular conditions during infection. Future studies using intracellular *S*. Typhimurium could provide an opportunity to explore how these differences in heat shock response influence pathogenicity.

In summary, these findings demonstrate that genome-scale experimental data can enhance our understanding of binding regions and their functionality, providing essential information for transcriptional regulation and genomic analysis of *S*. Typhimurium. In the future, a systems biology approach could elucidate complex TRNs, including various TFs and sigma factors, thereby allowing for a more accurate characterization of transcriptional impacts under infection-relevant environments [60,61].

## Method and materials

### Bacterial strains, media, and growth conditions

All strains used in this study are *S*. Typhimurium 14028s and its derivatives, knock-out strain and a myc-tagged strain. For ChIP-mini experiment of RpoH, the *S*. Typhimurium *rpoH*-8myc knock-in strain was generated as described previously [62]. The myc-tagged strain *S*. Typhimurium *rpoH*-8myc showed no growth change compared to the wild-type strain, indicating the myc epitope did not change the binding activity of RpoH. For expressions profiling by RNA-seq, deletion mutant *ΔrpoS* was constructed by λ red-mediated site-specific recombination system [63]. M9 minimal media was also supplemented with 1 ml trace element solution (100x) containing 1 g EDTA, 29 mg ZnSO_4_·7H_2_O, 198 mg MnCl_2_·4H_2_O, 254 mg CoCl_2_·6H_2_O, 13.4 mg CuCl_2_ and 147 mg CaCl_2_ [64]. For ChIP-mini and RNA-seq experiments, Glycerol stocks of *S*. Typhimurium strains were inoculated into M9 minimal media and cultured overnight at 37°C with constant agitation. Cultures were then diluted 1:100 into 50 mL of fresh minimal media and cultured at 37°C to mid- exponential phase (OD_600_ ≈ 0.6) before harvest. For heat-shock experiments, *S*. Typhimurium strains were grown to mid-exponential phase at 37°C. and half of the culture was used as a control, while the remaining culture was transferred into pre-warmed (50°C) media and incubated in a 42°C water bath with constant agitation for 15 min [5].

### ChIP-mini experiment

A low-input ChIP-exo (ChIP-mini) experiment was performed following the procedures previously described [31]. In brief, to identify sigma factors binding maps *in vivo*, we isolated the DNA bound to RpoD and RpoS from 5ml of formaldehyde cross-linked *S*. Typhimurium wild-type cells from the same flask. For DNA bound to RpoH, we used 5 ml of *S*. Typhimurium *rpoH*-8myc cells, which were cross-linked with formaldehyde. To isolate DNA-sigma factor complex, chromatin immunoprecipitation (ChIP) was performed with the antibodies that specifically recognize myc tag (9E10, Santa Cruz Biotechnology) or RpoD (2G10, Biolegend), and RpoS (1RS1, Biolegend) subunits of RNA polymerase complex, respectively [5,31]. Next, Dynabeads Pan Mouse IgG magnetic beads (Invitrogen) was used to isolate DNA-sigma factor complex bound to antibodies and followed by stringent washings as described previously [5]. ChIP materials (chromatin-Dynabeads) were used to perform on-bead enzymatic reactions using the ChIP-mini method, with an optimized volume of reagents [31,65,66]. Briefly, sheared chromatin-bead DNA was first repaired using the NEBNext End Repair Module (New England Biolabs). This step was followed by the addition of a single dA overhang and ligation of the first adaptor (5’-phosphorylated) using the dA-Tailing Module and NEBNext Quick Ligation Module (New England Biolabs), respectively. Nick repair was performed with the PreCR Repair Mix (New England Biolabs). Lambda exonuclease- and RecJf exonuclease-treated chromatin was then eluted from the beads, and protein–DNA cross-links were reversed by overnight incubation at 65°C. The resulting RNA- and protein-free DNA samples were used for primer extension and second adaptor ligation, incorporating the modifications previously described. The DNA samples, incubated for primer extension as previously described [66], were treated with the dA-Tailing Module (New England Biolabs) and NEBNext Quick Ligation Module (New England Biolabs) for second adaptor ligation. Subsequently, the DNA was purified using 1.0x HiAccuBead (Accugene) and enriched by polymerase chain reaction (PCR) using KAPA HiFi HotStart ReadyMix (KAPA Biosystems). The amplified DNA samples were purified again by 1.0x HiAccuBead (Accugene) and quantified using Qubit dsDNA HS Assay Kit (Life Technologies). quality of the DNA sample was checked by running Agilent High Sensitivity DNA Kit using Agilent 2100 Bioanalyzer (Agilent) before being sequenced using NextSeq550 (Illumina) in accordance with the manufacturer’s instructions. Each modified step was also performed in accordance with the manufacturer’s instructions. ChIP-mini experiments were performed in biological duplicate.

### RNA-seq expression profiling

For total RNA isolation, 3 ml of cells from mid-exponential phase culture and 6 ml of cells from heat shock treated culture were mixed with 6 ml and 12 ml RNAprotect Bacteria Reagent (Qiagen), respectively. Samples were immediately mixed and incubated at room temperature for 5 min, and then centrifuged at 5000xg for 10 min. The supernatant was decanted, any residual supernatant was removed by inverting the tube once onto a paper towel. Total RNA samples were then isolated using the RNeasy Plus Mini kit (Qiagen) according to the manufacturer’s instructions. The samples were quantified using a NanoDrop 1000 spectrophotometer (Thermo Scientific), and the quality of the isolated RNA was assessed with an RNA 6000 Pico Kit on an Agilent 2100 Bioanalyzer (Agilent). The ribosomal RNAs were removed with riboPOOLs for pan-prokaryote (siTOOLs). Following rRNA depletion, a paired-end, strand-specific RNA-seq library was constructed using the KAPA Stranded RNA-seq Library Preparation Kit (KAPA Biosystems) according to the manufacturer’s instructions. The resulting libraries were analyzed on an Agilent High Sensitivity DNA Kit using Agilent 2100 Bioanalyzer (Agilent) before being sequenced using NextSeq550 (Illumina) in accordance with the manufacturer’s instructions. All RNA-seq experiments were performed in biological duplicate.

### ChIP-mini output processing

Sequence reads generated from ChIP-exo were mapped onto the reference genome (NC_016856.1: genome, NC_016855.1: plasmid) using ChEAP (ChIP-exo analysis pipeline) including bowtie [67,68] with default options to generate SAM output files, these output files were converted to BAM and GFF files for further analysis. DEOCSU (DEep-learning Optimized ChIP-exo peak calling Suite), a novel machine learning-based ChIP-exo peak-calling suite pipeline, was used to define binding peak candidates from the biological duplicates [69]. To reduce false-positive peaks, those with a signal-to-noise ratio (S/N) less than 1.0 were removed. The noise level was set to the top 5% of signals at genomic positions because the top 5% make a background level in plateau and the top 5% intensities from each ChIP-mini replicate across conditions correlate well with the total number of reads. Genome-scale binding profiling data were visualized using MetaScope [69].

### Identification of differentially binding peaks

To normalize binding intensity and identify differentially binding peaks (DBPs) between control and heat shock conditions, DiffExo pipeline was used (https://github.com/SBML-Kimlab/DiffExo) [31]. The RPPM (Reads Per Peak per Million) value was conceived to normalize ChIP-mini sequencing datasets. The calculation of RPPM for peak *i* uses the following formula:

$$R{PPM}_{i}\;(Reads\;Per\;Peak\;per\;Million)=\frac{q_{i}}{l_{i}*\;\frac{\sum_{j}q_{j}}{10^{6}}}=\frac{q_{i}}{l_{i}*\;\sum_{j}q_{j}}*\;10^{6}$$

where *q*_*i*_ are raw sequencing read counts, *l*_*i*_ is ChIP-mini binding peak length, and ∑_*j*_*q*_*j*_ corresponds to the total number of mapping reads on the reference genome. Statistically, significant differentially binding sites were calculated using DESeq2, which adopted a negative binomial distribution-based model [70]. From the DESeq2 output, peaks with log_2_ fold change ≥ 1.0 and false discovery rate < 0.05 were considered as DBPs.

### Motif search and analysis

Sequence motif analysis for sigma-factors was performed using the MEME software suite [71]. For three sigma factors, sequences in binding sites were extracted from the reference sequence (NC_016856.1: genome, NC_016855.1: plasmid). For RpoD and RpoS, sequences were extended by 20 bp away from target genes, because only −10 box was found without that extension.

### RNA-seq output processing

Sequence reads generated from RNA-seq were mapped onto the reference genome (NC_016856.1: genome, NC_016855.1: plasmid) using bowtie [67] with the maximum insert size of 1,000 bp, and 2 maximum mismatches after trimming 3 bp at 3’ ends. SAM files were generated from bowtie mapping. To compare expression changes according to heat shock or genetic deletion mutants, featureCount and DESeq2 was used to calculate transcripts per million (TPM) value and differential expression [70]. From DESeq2 output, genes with differential expression with absolute value of log_2_ fold change ≥1.0 and false discovery rate <0.05 were considered differentially expressed genes (DEGs). Genome-scale data were visualized using MetaScope [69].

### COG functional enrichment

Up-regulated or down-regulated DEGs were categorized according to their annotated clusters of orthologous groups (COG) category using BPGA, an ultra-fast pan-genome analysis pipeline [72]. Functional enrichment of COG categories in target genes was determined by performing a hypergeometric test, and a *p*-value <0.05 was considered significant.

### Inferring activity of iModulons

To decompose transcriptional impact of heat stress, PyModulon Python package [73] and strain specific RNA-seq compendium for *S*. Typhimurium 14028s [33] was utilized to infer activity of iModulons. Four expression profiles, generated under both control and heat shock conditions, were normalized to log_2_(TPM) to construct a new dataset table. This table was applied to the RNA-seq compendium for 14028s, confirming the overlap of genes between the ICA dataset and the new dataset, as non-overlapping genes were ignored. Next, among expression profiles, two expression profiles, generated under control conditions, were used to be centered as a reference condition by averaging the replicates. Finally, inferring relative iModulon activities of new dataset was conducted utilizing PyModulon [73]. In addition, a core RNA-seq compendium containing all 534 expression profiles was used to confirm the relative change in activity of the fnr-1 iModulon under anaerobic growth or shock conditions.

## Supporting information

S1 Table Differentially expressed genes (DEGs) due to heat shock. (XLSX)

S2 Table Total binding sites of RpoD-family sigma factors. (XLSX)

S3 Table RpoD, RpoS, and RpoH heat shock sigmulons. (XLSX)

S4 Table RpoS sigmulon under control and heat shock conditions. (XLSX)

S1 Fig Heat shock-dependent transcriptome analysis of *S*. Typhimurium 14028s using iModulons. Activity of iModulons in *S*. Typhimurium 14028s RNA-seq compendium under heat shock conditions.
(TIF)

S2 Fig The genome-wide binding profiles for RpoD, RpoS, and RpoH under both control and heat shock conditions. Overlaps between RpoD and RpoS or RpoH binding sites. In response to heat shock, the number of RpoS binding sites overlapping with RpoD increased from 889 to 937, and RpoH binding sites overlapping with RpoD increased from 52 to 53. (The grey box indicates events where one binding site is overlapped by two or more binding sites of another sigma factor.) (B) Number of bindings of three RpoD-family sigma factors in regulatory and non-regulatory regions. (C) Binding intensities of regulatory regions and non-regulatory regions were calculated. Non-regulatory bindings have weaker intensities (rank sum test *p*-value < 0.05). (D) Percentage of up or down-regulated DEGs in sigma factor binding genes. Three sigma factors predominantly bind upstream of upstream of transcription start site (TSS) facilitating transcription initiation in response to heat shock.
(TIF)

S3 Fig Reconstruction of RpoS regulon under both control and heat shock conditions. (A) The scatter plot illustrates the relationships between intensity and transcript expression levels related to RpoD and RpoS binding in response to heat shock. The change in binding intensity of RpoD and RpoS showed similar trends. When the binding intensity of the two sigma factors increased, the expression level tended to be up-regulated, whereas when the binding intensity decreased, the expression level tended to be down-regulated. (B) Genome-wide transcriptional regulatory roles of RpoS. Majority of RpoS regulon genes were activated by RpoS, and the number of regulon genes were significantly expanded by heat shock. (C) Change in intensity of RpoS and RpoD overlapping binding sites from RpoS regulon. Binding intensity of RpoS was increased in response to heat shock, while RpoD did not show notable increase in intensity, indicating that RpoS increased its binding upstream of TSS to compete with RpoD.
(TIF)

S4 Fig The mRNA expression level of heat shock proteins in response to heat shock. Three asterisks denote significant expression change (log_2_ fold change ≥ 1.0 and false positive rate <0.001).
(TIF)

S5 Fig ChIP-mini binding profiles of RpoD upstream of NADH dehydrogenase transcription unit. The RpoD binding site, indicated by red dotted line, corresponds with the TSS of the NADH dehydrogenase transcription unit (TU).
(TIF)

S6 Fig Mechanism of RpoS activation by ppGpp in response to environmental stimuli. (A) Under conditions of nutrient abundance, phosphorylated RssB attaches to RpoS to facilitate the degradation of the RssB-RpoS complex by ClpXP. On the other hand, ppGpp is produced in response to environmental stimuli, inducing the expression of RssB binding protein gene, *iraP* or *iraD*. Induction of RssB binding proteins results in RpoS under phosphate-limiting conditions (*iraP*) or steady state (*iraD*). (B) The mRNA expression levels of genes involved in the activation of RpoS with ppGpp under control and heat shock conditions. Notably, expression level of *iraP* was significantly up-regulated, suggesting that RpoS activation mechanism in response to heat stress is similar to phosphate limiting stress. Three asterisks denote significant expression change (log_2_ fold change ≥ 1.0 and false positive rate <0.001).
(TIF)

S7 Fig ChIP-mini binding profiles of RpoD upstream of iron metabolism genes. Binding intensity of RpoD upstream of iron metabolism genes decreased in response to heat shock. Especially, intensity of RpoD bindings upstream of *fepE*, *fhuACDB*, *feoABC*, and *exbBD* significantly reduced (LFC <-1.0 and false positive rate <0.05).
(TIF)

S8 Fig ChIP-mini binding profiles of RpoS upstream of iron metabolism genes. Binding intensity of RpoS upstream of sitABCD significantly decreased under heat shock conditions (LFC <-1.0 and false positive rate <0.05). In addition, intensity of RpoS bindings upstream of *ftnB* and *sufABCDSE* increased.
(TIF)

S9 Fig Heat map of sigma factors binding and expression changes for iModulons genes related in arginine and nucleotide biosynthesis in response to heat shock. The upper three stacks denote the binding of each sigma factor, and the bottom stack indicates the relative expression of genes between heat shock (42°C) and control conditions (37°C).
(TIF)

S10 Fig Genome-wide sigma factors binding and expression profiles of *Salmonella* pathogenic island-2 (SPI-2) and SPI-2 effector genes. (A) mRNA expression changes in six SPIs in response to heat shock. (B) The mRNA expression levels of SPI-2 and SPI-2 effector genes involved in each iModulon (SPI2 and SPI2-T3SS) under control and heat shock conditions. Three asterisks denote significant expression change (log_2_ fold change ≥ 1.0 and false positive rate <0.001). Two asterisks illustrate significant expression change (log_2_ fold change ≥ 1.0 and false positive rate <0.05). (C) Heat map of sigma factors binding and expression changes for SPI-2 and SPI-2 effector genes in response to heat shock. (The upper three stacks denote the binding of each sigma factor, and the bottom stack indicates the relative expression of genes between heat shock (42°C) and control conditions (37°C).
(TIF)

S11 Fig Comparison of RpoS binding sites upstream of *aceBA* in *E*. *coli* K-12 MG1655 and *S*. Typhimurium 14028s. (TIF)
