# Supplementary figures and images for: Unveiling the novel regulatory roles of RpoD-family sigma factors in Salmonella Typhimurium heat shock response through systems biology approaches
Source: PLoS Genet. 2024 Oct 29;20(10):e1011464. doi: 10.1371/journal.pgen.1011464 (PMC11548764; doi:10.1371/journal.pgen.1011464)

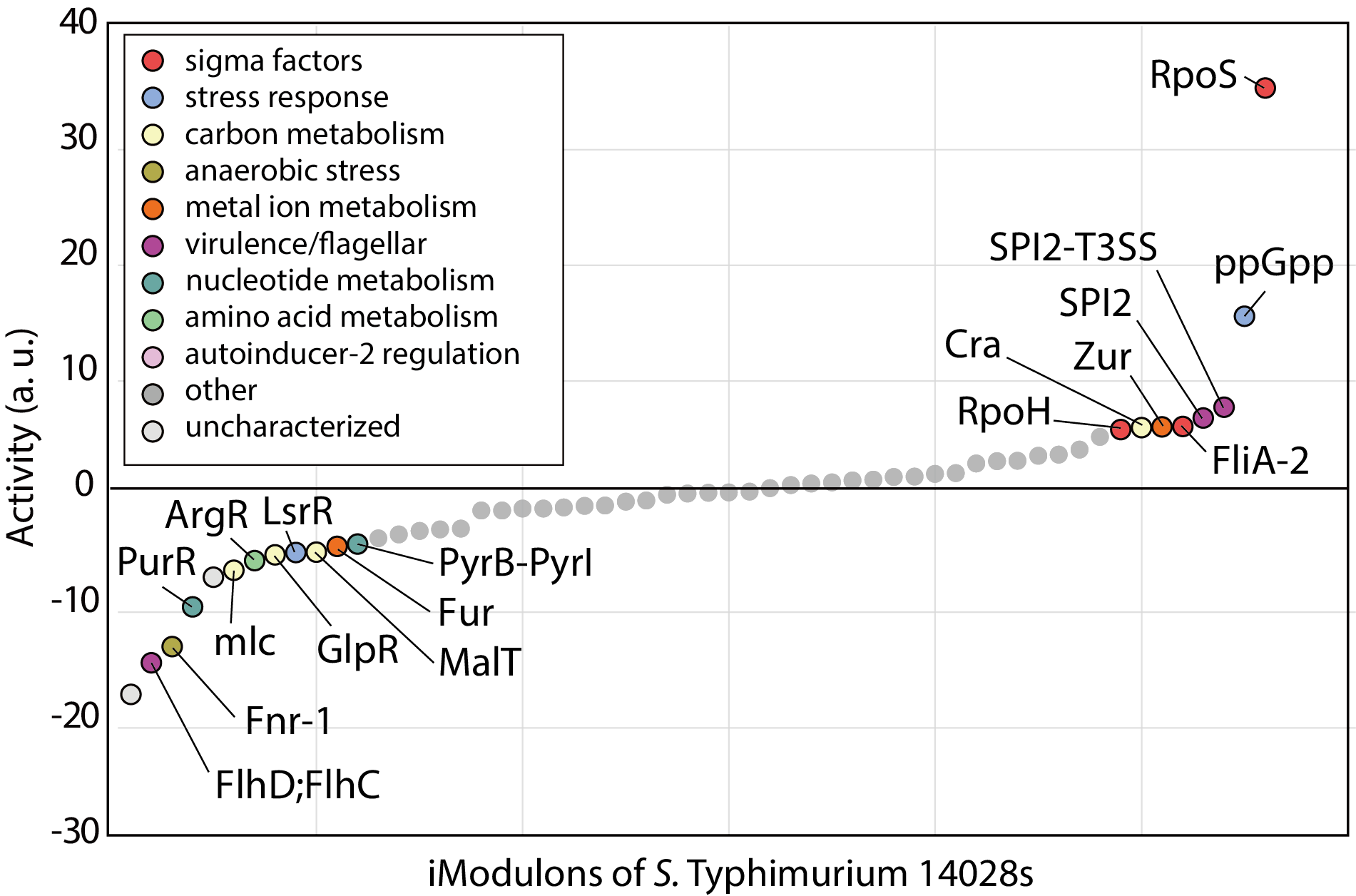

Supplement: S1 Fig — Activity of iModulons in S. Typhimurium 14028s RNA-seq compendium under heat shock conditions. (TIF) [file pgen.1011464.s005.tif]

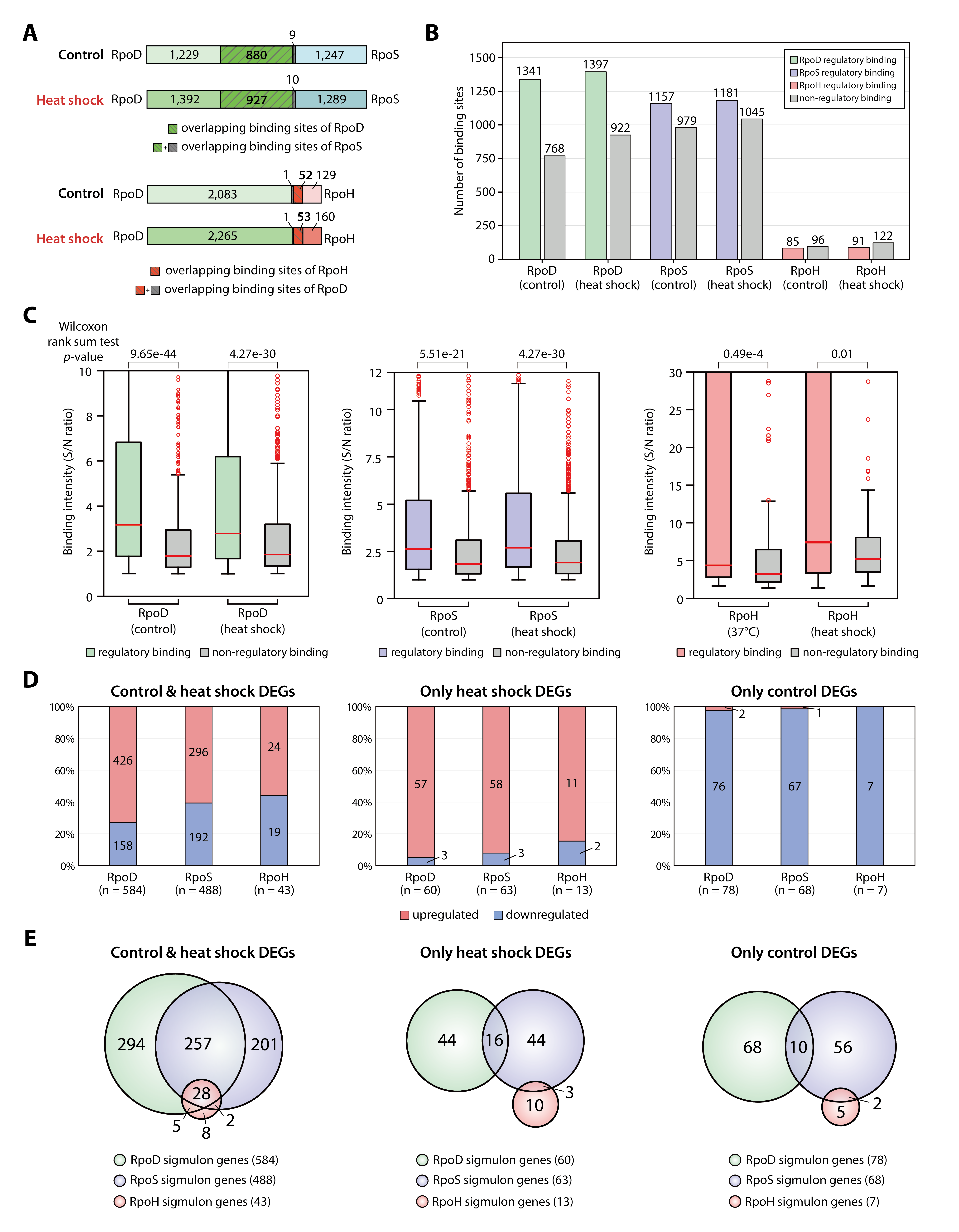

Supplement: S2 Fig — Overlaps between RpoD and RpoS or RpoH binding sites. In response to heat shock, the number of RpoS binding sites overlapping with RpoD increased from 889 to 937, and RpoH binding sites overlapping with RpoD increased from 52 to 53. (The grey box indicates events where one binding site is overlapped by two or more binding sites of another sigma factor.) (B) Number of bindings of three RpoD-family sigma factors in regulatory and non-regulatory regions. (C) Binding intensities of regulatory regions and non-regulatory regions were calculated. Non-regulatory bindings have weaker intensities (rank sum test p-value < 0.05). (D) Percentage of up or down-regulated DEGs in sigma factor binding genes. Three sigma factors predominantly bind upstream of upstream of transcription start site (TSS) facilitating transcription initiation in response to heat shock. (TIF) [file pgen.1011464.s006.tif]

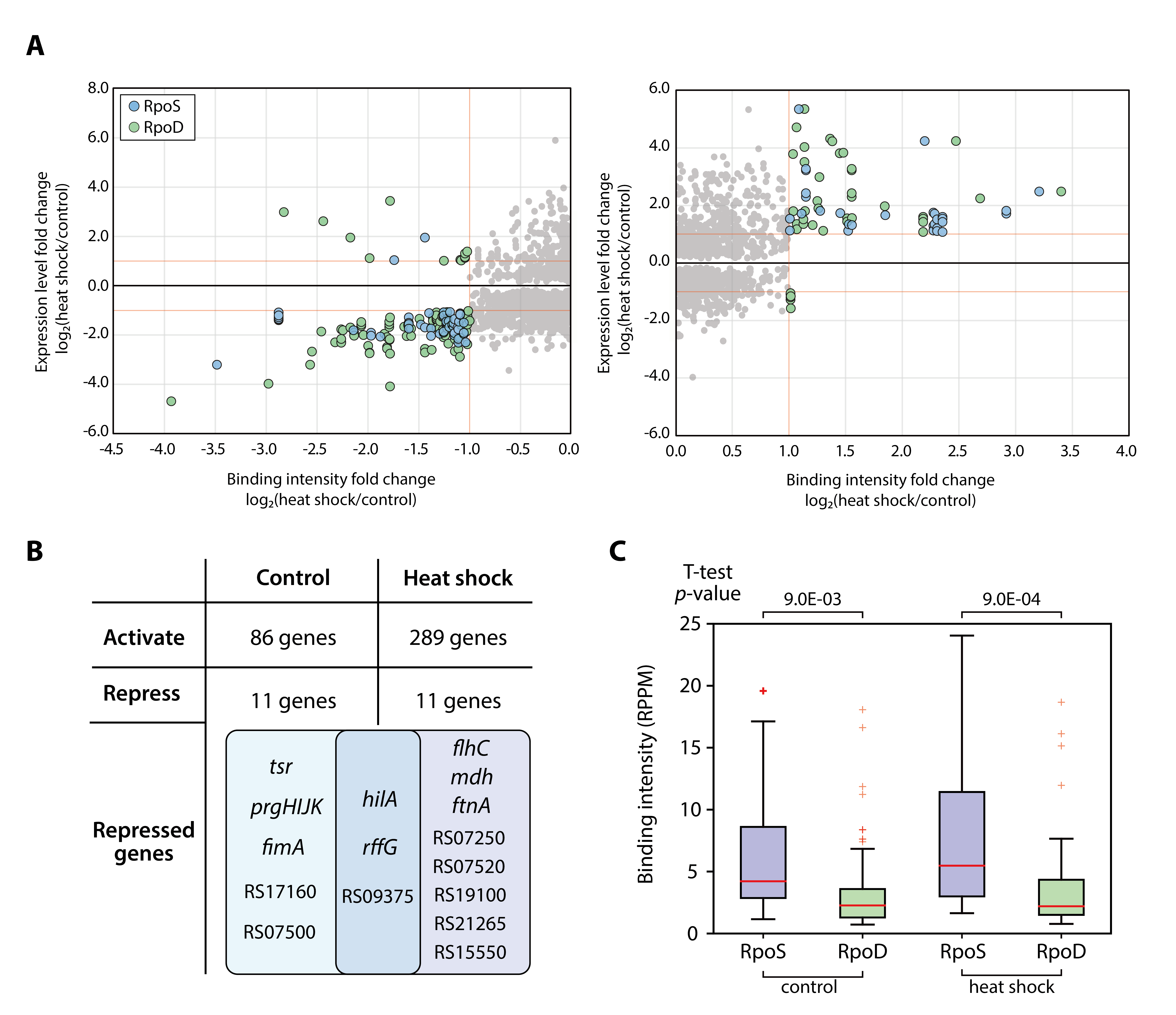

Supplement: S3 Fig — (A) The scatter plot illustrates the relationships between intensity and transcript expression levels related to RpoD and RpoS binding in response to heat shock. The change in binding intensity of RpoD and RpoS showed similar trends. When the binding intensity of the two sigma factors increased, the expression level tended to be up-regulated, whereas when the binding intensity decreased, the expression level tended to be down-regulated. (B) Genome-wide transcriptional regulatory roles of RpoS. Majority of RpoS regulon genes were activated by RpoS, and the number of regulon genes were significantly expanded by heat shock. (C) Change in intensity of RpoS and RpoD overlapping binding sites from RpoS regulon. Binding intensity of RpoS was increased in response to heat shock, while RpoD did not show notable increase in intensity, indicating that RpoS increased its binding upstream of TSS to compete with RpoD. (TIF) [file pgen.1011464.s007.tif]

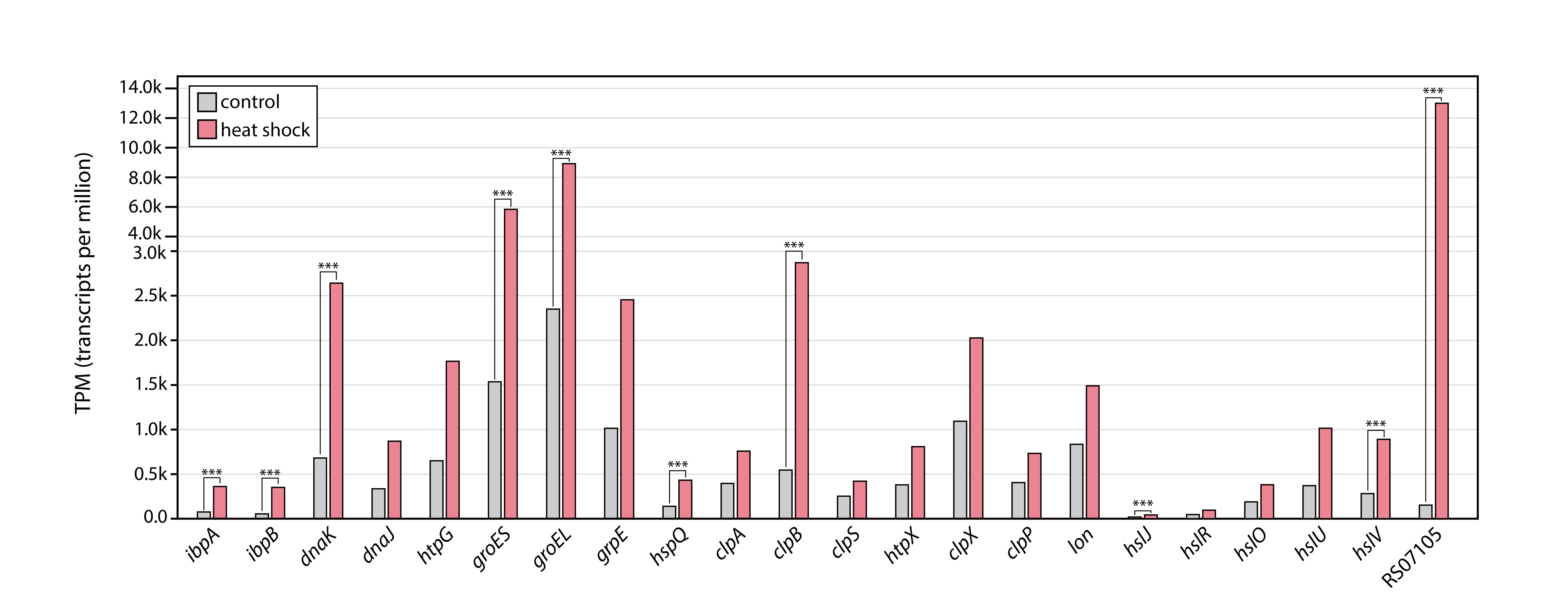

Supplement: S4 Fig — Three asterisks denote significant expression change (log2 fold change ≥ 1.0 and false positive rate <0.001). (TIF) [file pgen.1011464.s008.tif]

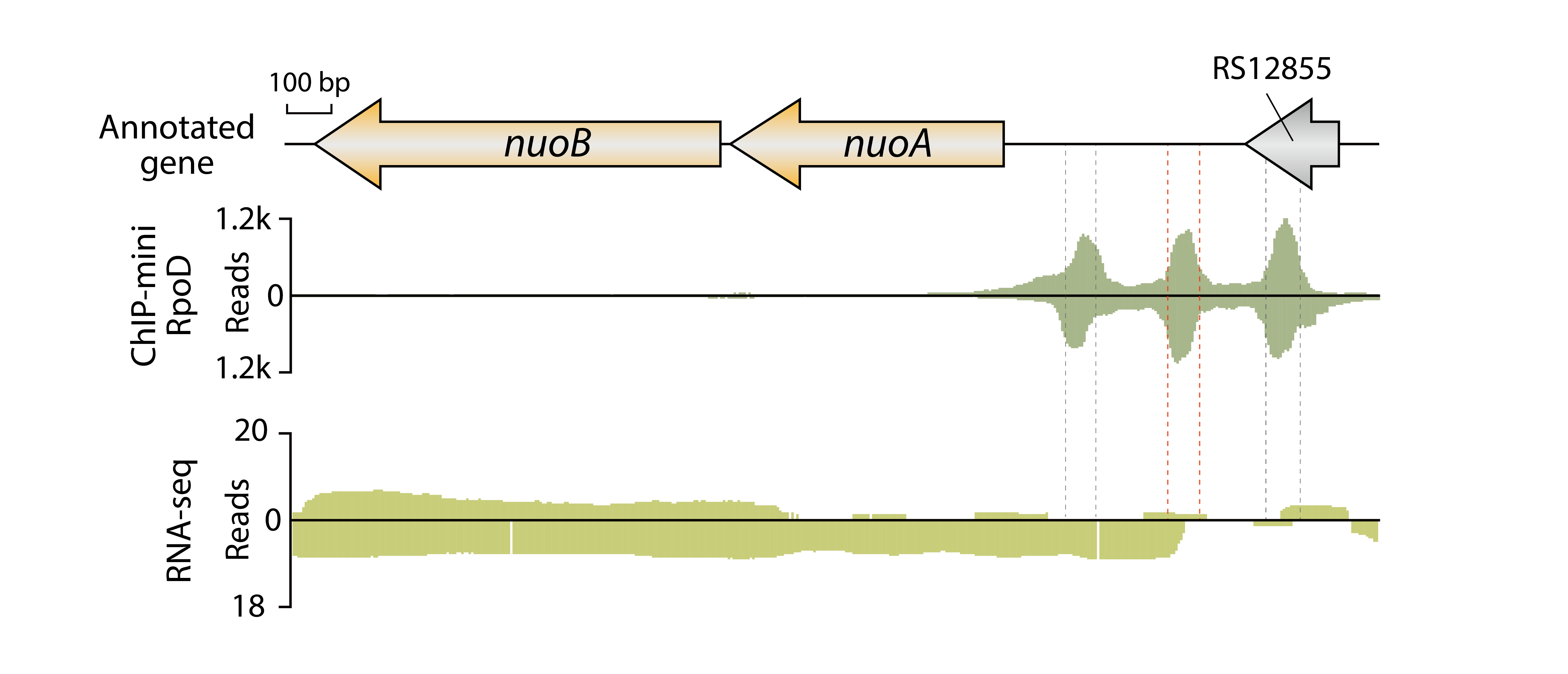

Supplement: S5 Fig — The RpoD binding site, indicated by red dotted line, corresponds with the TSS of the NADH dehydrogenase transcription unit (TU). (TIF) [file pgen.1011464.s009.tif]

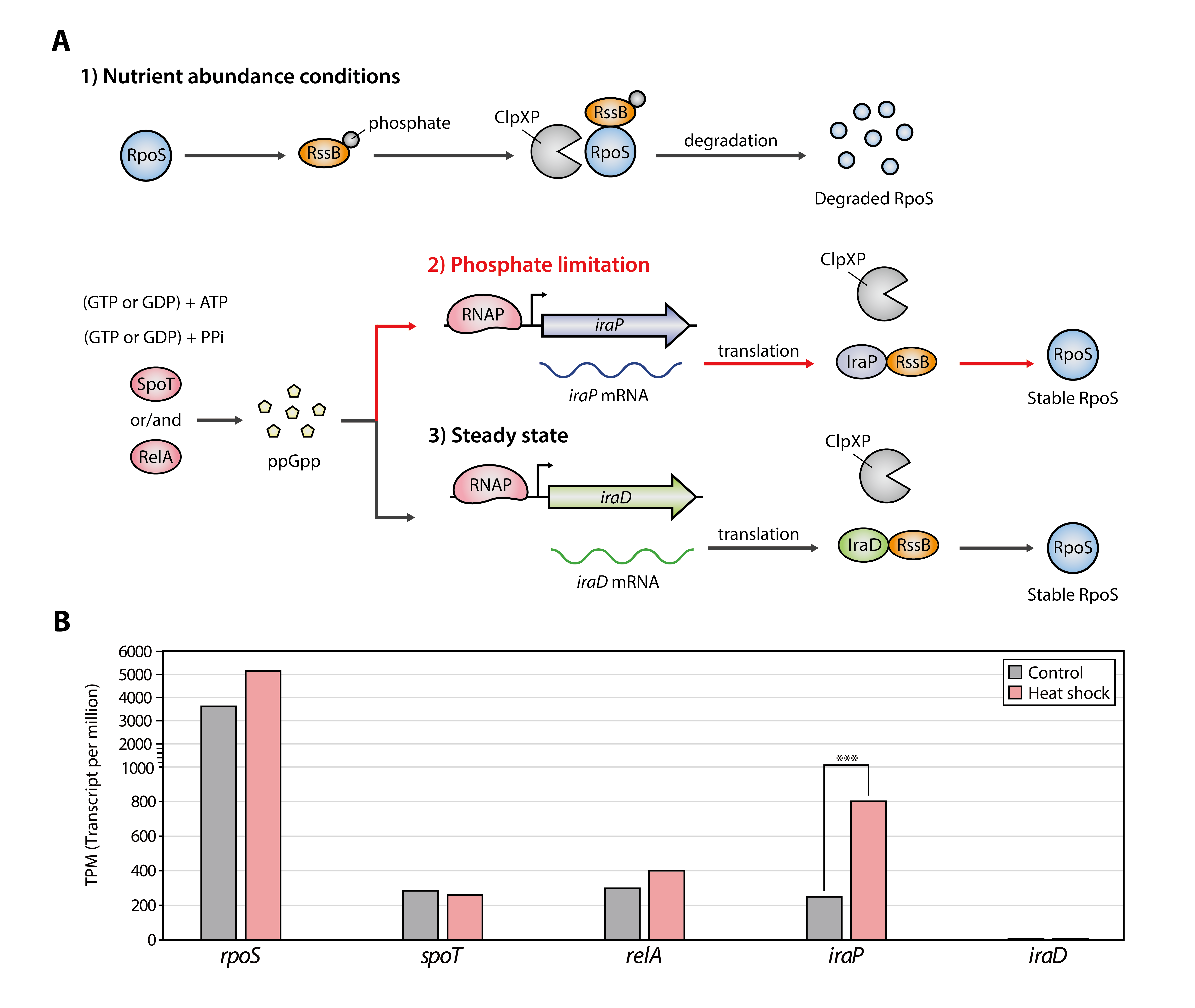

Supplement: S6 Fig — (A) Under conditions of nutrient abundance, phosphorylated RssB attaches to RpoS to facilitate the degradation of the RssB-RpoS complex by ClpXP. On the other hand, ppGpp is produced in response to environmental stimuli, inducing the expression of RssB binding protein gene, iraP or iraD. Induction of RssB binding proteins results in RpoS under phosphate-limiting conditions (iraP) or steady state (iraD). (B) The mRNA expression levels of genes involved in the activation of RpoS with ppGpp under control and heat shock conditions. Notably, expression level of iraP was significantly up-regulated, suggesting that RpoS activation mechanism in response to heat stress is similar to phosphate limiting stress. Three asterisks denote significant expression change (log2 fold change ≥ 1.0 and false positive rate <0.001). (TIF) [file pgen.1011464.s010.tif]

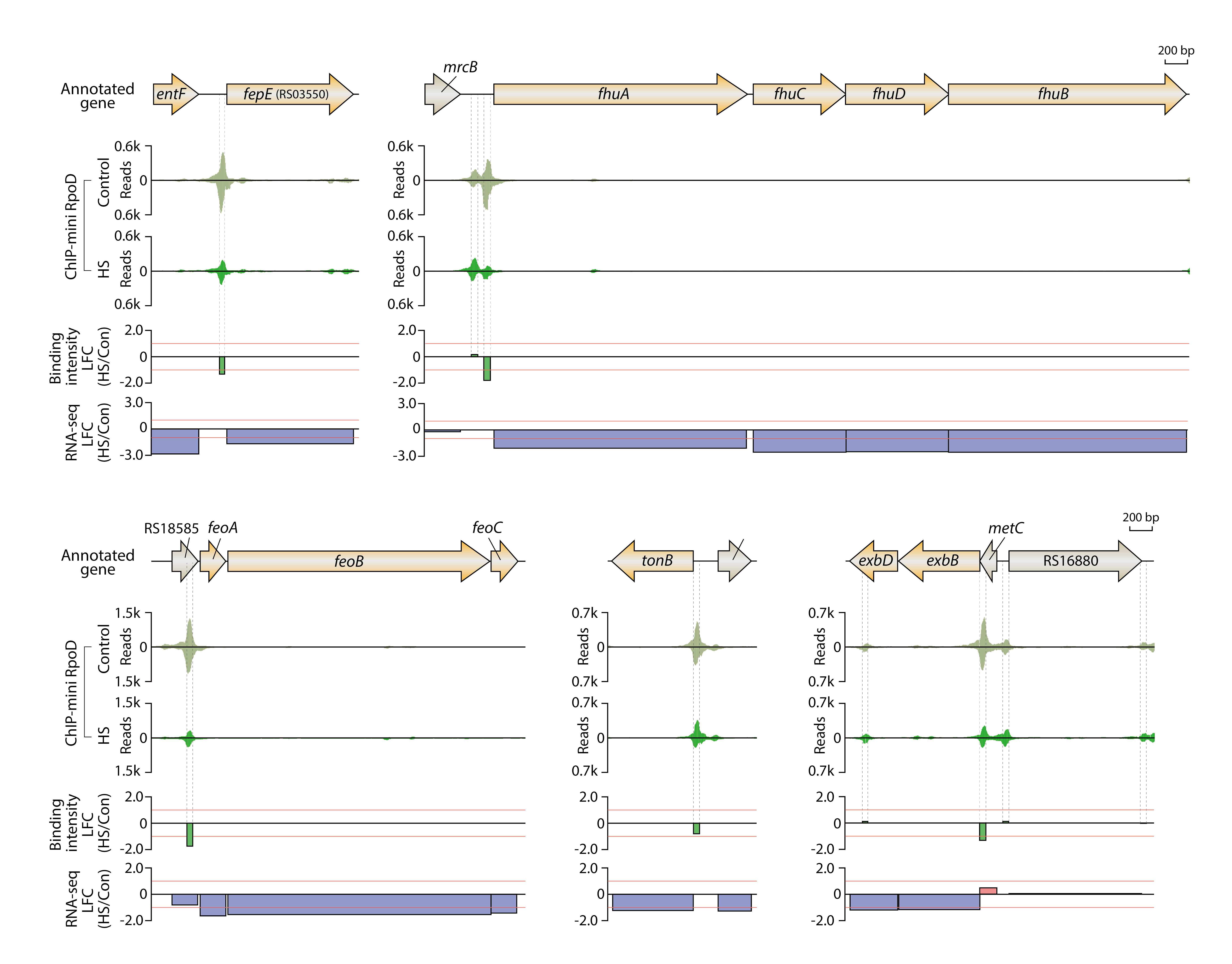

Supplement: S7 Fig — Binding intensity of RpoD upstream of iron metabolism genes decreased in response to heat shock. Especially, intensity of RpoD bindings upstream of fepE, fhuACDB, feoABC, and exbBD significantly reduced (LFC <-1.0 and false positive rate <0.05). (TIF) [file pgen.1011464.s011.tif]

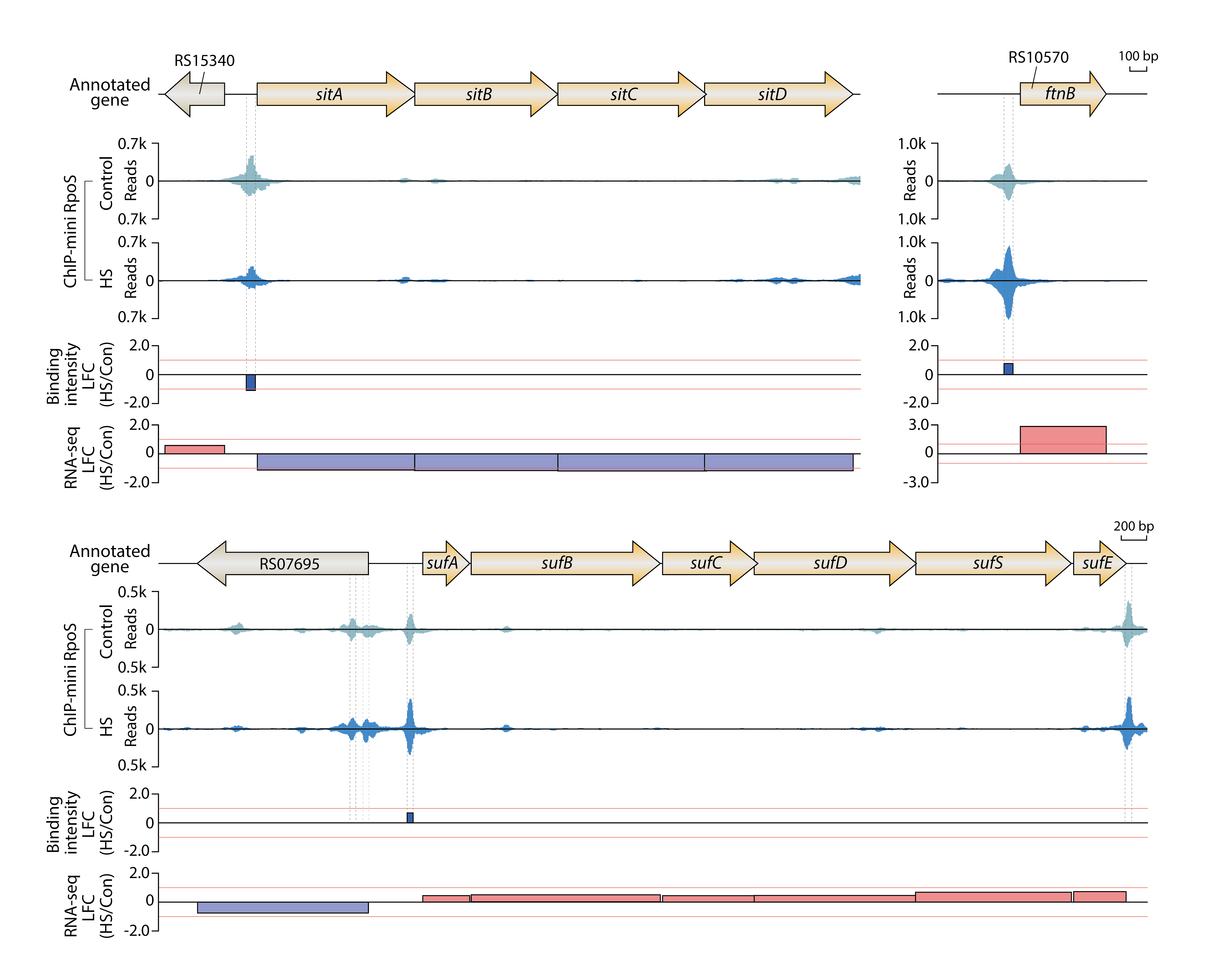

Supplement: S8 Fig — Binding intensity of RpoS upstream of sitABCD significantly decreased under heat shock conditions (LFC <-1.0 and false positive rate <0.05). In addition, intensity of RpoS bindings upstream of ftnB and sufABCDSE increased. (TIF) [file pgen.1011464.s012.tif]

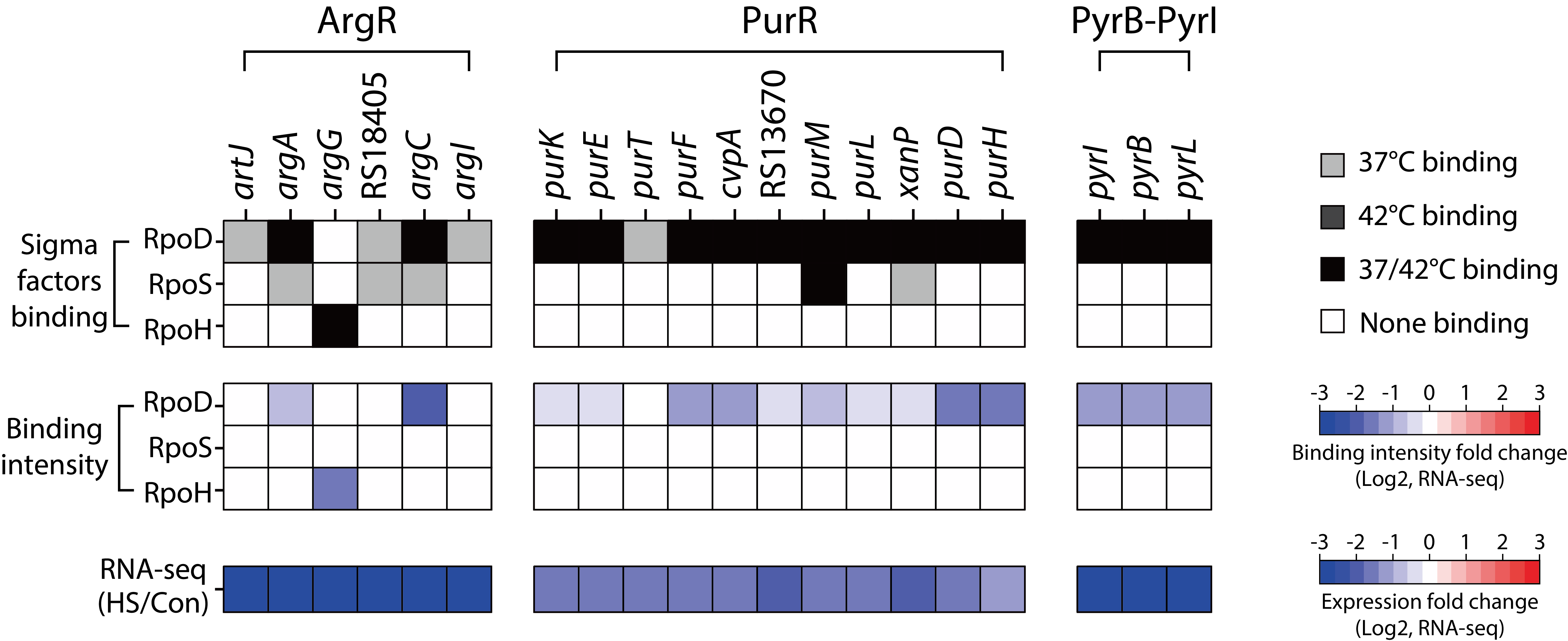

Supplement: S9 Fig — The upper three stacks denote the binding of each sigma factor, and the bottom stack indicates the relative expression of genes between heat shock (42°C) and control conditions (37°C). (TIF) [file pgen.1011464.s013.tif]

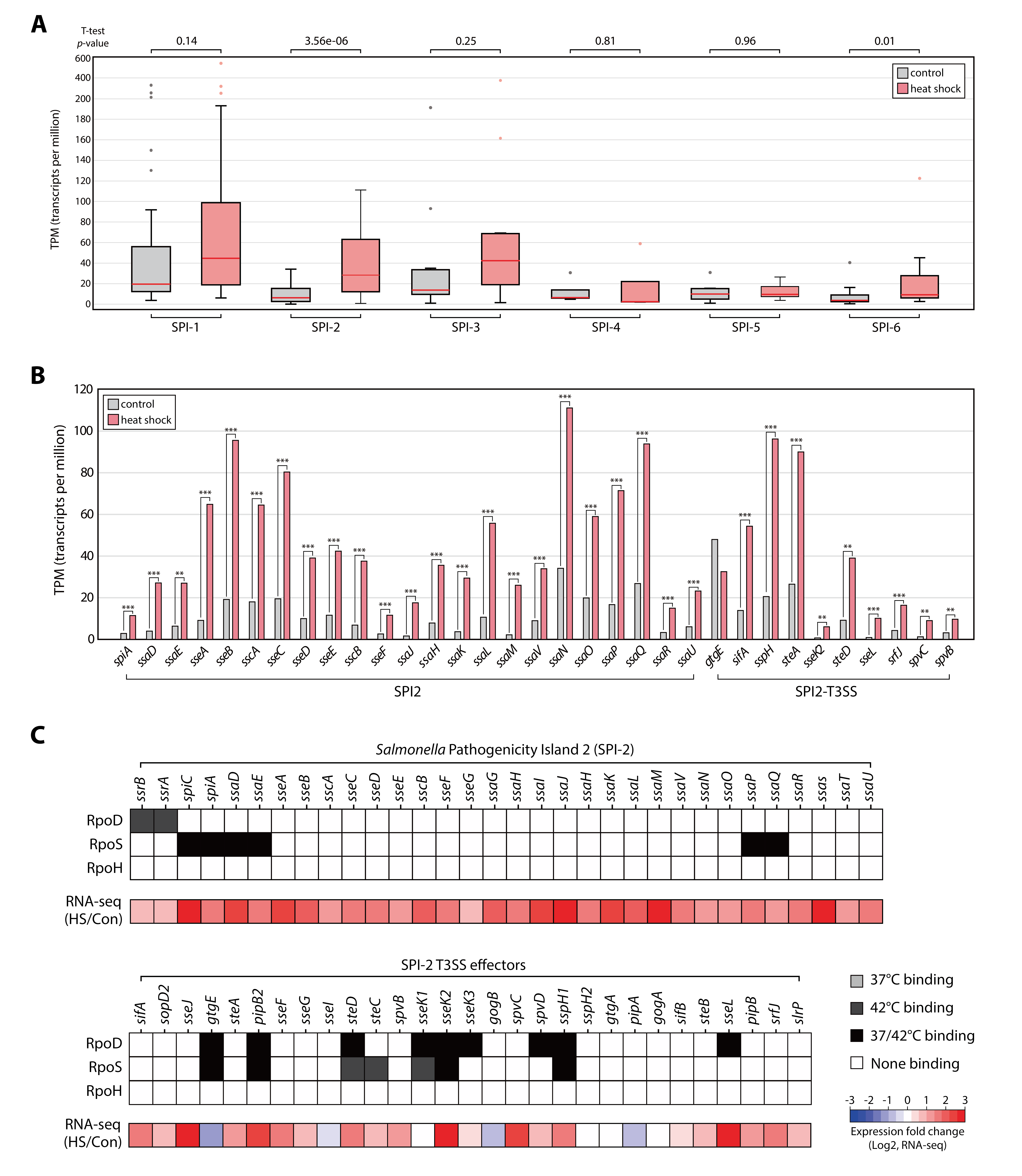

Supplement: S10 Fig — (A) mRNA expression changes in six SPIs in response to heat shock. (B) The mRNA expression levels of SPI-2 and SPI-2 effector genes involved in each iModulon (SPI2 and SPI2-T3SS) under control and heat shock conditions. Three asterisks denote significant expression change (log2 fold change ≥ 1.0 and false positive rate <0.001). Two asterisks illustrate significant expression change (log2 fold change ≥ 1.0 and false positive rate <0.05). (C) Heat map of sigma factors binding and expression changes for SPI-2 and SPI-2 effector genes in response to heat shock. (The upper three stacks denote the binding of each sigma factor, and the bottom stack indicates the relative expression of genes between heat shock (42°C) and control conditions (37°C). (TIF) [file pgen.1011464.s014.tif]

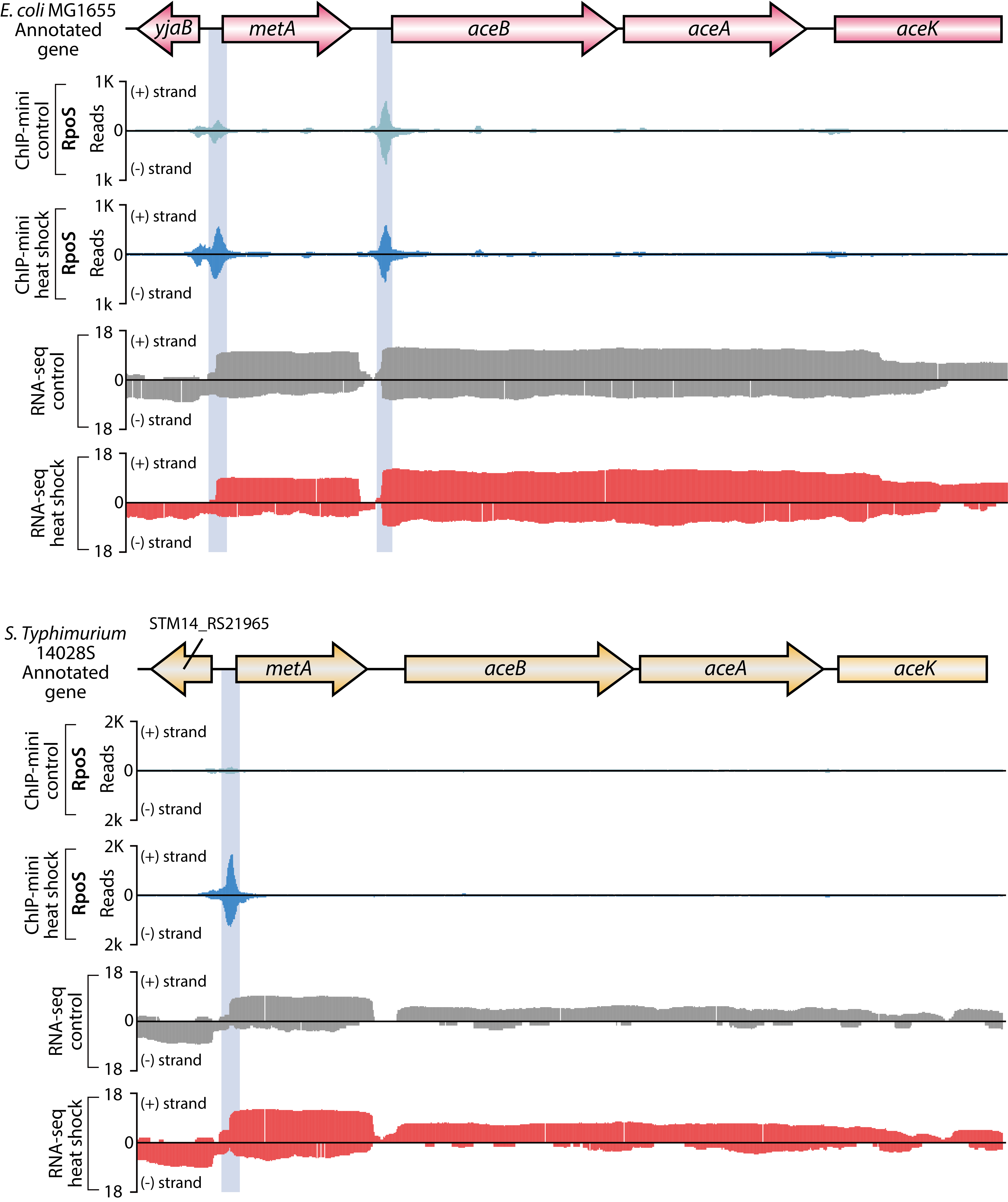

Supplement: S11 Fig — (TIF) [file pgen.1011464.s015.tif]
